# The population of Veneto faced with technological consumption of music during the pandemic

**DOI:** 10.3389/fpsyg.2024.1329531

**Published:** 2024-07-08

**Authors:** María del Valle De Moya Martínez, Alfredo Segura Tornero, Cedric Marc Alain Casabon, José Antonio Hernández Bravo

**Affiliations:** ^1^Faculty of Education, Department of Didactics of Physical, Artistic and Music Education, University of Castilla La Mancha, Albacete, Spain; ^2^Faculty of Education, Department of Modern Languages, University of Castilla La Mancha, Albacete, Spain

**Keywords:** social media, musical habits, music and everyday life, COVID-19, youth music consumption, online music broadcasting

## Abstract

**Objective:**

This work is the result of one of the lines of research opened in the LabinTic group (UCLM) and a stay in the city of Padua, in the Italian Veneto area, in 2022. After observing the side effects in Italy as a result of COVID-19 and the Great Lockdown, we wanted to know in more depth some aspects related to how the people of Veneto used IT as an important weapon to fight against forced isolation and, if possible, compare this with other similar studies performed in Castilla-La Mancha.

**Method:**

To achieve this objective, an *ad hoc questionnaire* was designed and validated through expert judgment. This questionnaire was administered to a random probabilistic sample by disseminating it through social media and email using the “snowball” method. A total of 338 people who comprised the population sample of the study (*n* = 338) were obtained. After analyzing the data through SPSS 28, using a mixed methodology considering qualitative and quantitative aspects, the great influence that IT had among the Italian population during the lockdown common aspects of their everyday lives and incorporating some acquired habits (listening to music, for example) into their usual life routines after the return to normality was determined among other features and assessments. In summary, the text highlights how the pandemic influenced music consumption, the popularity of different music genres, the role of social media, and the enduring importance of the music itself in people’s lives. It suggests that music remains a source of comfort and entertainment in challenging times.

## 1 Introduction

Research on technology has incorporated users into its studies, showing that their creativity influences technological development in all of its processes, from its original design to its implementation. The traditional approach of man and machine has been replaced by that of user and use, with studies about consumption as a cultural activity proliferating. These analyses study the use of users with respect to technology: how they consume, modify, customize, reconfigure, or resist technological development; in essence, how the power and influences of technology define and transform the everyday activities of users (Oudshoorn and Pinch, 2003).

The relationship between consumption, technology, and music is a specific area. Consumption is a basic activity in everyday life that is useful to articulate the identity, culture, and even history of social groups. In the current era, it has acquired a strong prominence, although not exempt from negative connotations, something that contrasts with the idea of “colorful consumption,” which understood it as a fashionable social function at the end of the 19th century. After the economic increase and improvements in quality of life produced after World War II, the consumption of goods gained social prominence and became a distinctive aspect of Western historical evolution.

The act of consuming is a basic fact that articulates the identity, history, and culture of social groups. In today’s society, the value of some products is related, in large part, to creative processes, both cultural and symbolic, so that the possibility of consuming affects spheres of social life. Thus, we speak of cultural consumption as a distinctive social behavior.

Magaudda (2012) reflects on the transformation of contemporary social life and the role played by technology in these changes. The author considers that the use and consumption of objects and technology is a privileged form of active social participation and analyzes the incidence and relevance of technical devices and technology in everyday life.

A school of thought considers that the possession of objects and goods constitutes a non-verbal social communicative universe, an information system, which is a common code in the creation of memberships and groups and establishes symbolic limits between social groups. However, social science research has shown little interest in analyzing the role of objects (and, by extension, technologies) in shaping society. The work of Moloch (2003), framed in what is known as the sociology of technology or science and technology studies (STS) (Magaudda, 2012, p. 26), stands out.

The phase of use and consumption of technologies can therefore be considered, following a definition given by two of the leading scholars of the social uses of technologies, as a “culturally contested area, in which users, consumer associations, designers, producers, sellers, *policymakers*, and groups of intermediaries negotiate and give different and even conflicting meanings and uses to technologies” (Oudshoorn and Pinch, 2003, p. 24).

The relations between technologies and society are complex because they result from a constant mutual adaptation between both spheres. A good example of this interrelation between individuals, society, and technology is social media, which has acquired an undisputed role in the current everyday life of a large part of the population, especially among younger groups.

Further focusing on the links and interrelations between technology and people highlights the issue that has given rise to this study: the connections between social media and habits and forms of music consumption, in an almost constant process of changes that affect even the music industry itself. This is because virtual platforms numerically increase their receiving audience. It is a means used by new artists to make themselves known, and it effectively promotes musical works. These allow the user to explore an immense musical production to discover new styles, genres, and works.

Social media is “a space created virtually to facilitate interaction between people” (Hütt Herrera, 2012, p. 123). De Aguilera-Moyano et al. (2010) state that the human relationships established through them are varied and complex: of closeness or friendship, with different degrees of action and interrelationship among users, and with great possibilities to deal with various topics (professional, leisure, informative, communicative, critical, everyday, …).

The influence of social media on society and the individual today is a widely analyzed topic (Wortman, 2019). However, it is also the main objective of this research, seeking to answer questions such as: which musical artistic use do users grant to social media [investigate and discover new styles, composers, musical talents, other music (ethnic, urban, past, among others)]? Does the use of social media affect people’s everyday habits? and Can social media influence the construction of the identity of social groups (e.g., young people)?

Technological tools are also a gateway to all types of knowledge and social interaction, but this fact forces parents and teachers to educate the younger generations, the digital natives, with the correct training that allows them to make adequate technological and emotional use of them. They must transmit guidelines that allow them to use these conveniently to ensure that, in the future, they are responsible, proactive citizens involved in improving society and preserving the environment. Additionally, they can become a motivating incentive to discover new knowledge and a way of expressing creativity.

The enormous implosion of social media in the everyday lives of citizens has caused, among other issues, a strong change in the uses and musical customs of users. This is corroborated by digital apps such as *Instagram, TikTok, Spotify,* or *Youtube*, which have allowed free musical access to a large part of the population, increased the discovery of genres and styles, eras, composers, and performers, modified the forms of musical consumption, favored social relations by eliminating borders when sharing music on networks, developed the artistic and musical creativity of those who use them, and changed the market strategies of the music industry to adapt to the new environments of the digital age. Even some of these networks are a means of advertising and promoting all types of music (Álvarez Álvarez, 2022).

In another regard, determining the musical styles and genres preferred by users can be a factor that helps with self-knowledge, especially in the field of emotions.

Music is a human product that has maintained its importance in different human societies over time. Music consumption has been changing and evolving as much as music itself, giving rise to the music industry in the last century. The last years of the last century witnessed strong and rapid changes in musical uses thanks to technological innovations that allowed a more agile and cheaper musical consumption through the use of different devices and virtual platforms, allowing an increasingly numerous sector of listeners almost instantaneous access to all types of musical content. Proof of this is the evolution of the live concert in an auditorium or outdoors, recorded or broadcast music (gramophone, radio, vinyl record, TV, cassette, CD, MP3, Internet, …).

Since time immemorial, music has been the traveling companion of human beings in their vital, individual, and community evolution (del De Moya Martínez et al., 2014; Fernández, 2018). It connects individuals by removing temporal and geographical boundaries, creating social connections, and removing cultural barriers and personal differences.

Throughout history, music has expressed feelings and emotions (Moscoso Córdova et al., 2021), communicated ideas and messages (Torres Lazcano, 2016), and been aesthetically and spiritually connected with the interior of the person (Fernández, 2018). This importance of the relationship between music and the human being is highlighted by the fact that music stimulates various cognitive processes (Soria Urios et al., 2011), contributes to the development of language (Llanga Vargas and Insuasti Cárdenas, 2019), enhances social interaction, expresses beauty, and transmits spirituality (del De Moya Martínez et al., 2014). Music suggests and communicates to the listener a wide and varied range of feelings and emotions (euphoria, joy, melancholy, sadness, happiness, etc.) and is capable of affecting moods. It brings calmness and security in childhood; touches, saddens, or rejoices and stimulates; awakens memories and evokes memories of the past; establishes pathways of personal relationships (Fustinoni, 2021); provides pleasure and distraction; shares emotions and thoughts; and helps to feel better and be more creative (Fernández, 2018); promotes cognitive skills and emotional education; improves self-esteem and relieves psychic situations, improving the quality of everyday life (Mosquera Cabrera, 2013).

Music is with us every step we take, it’s a vital experience. There is nothing more human than emotions and as stated in this work, music can provide the human being with the emotional and affective balance necessary for their vital flow. In addition, music is good for health and, regardless of culture, is an inherent element of the human being (del De Moya Martínez et al., 2023, p. 34).

Technology has allowed free access from anywhere in the world to the vast musical universe (Marquez, 2011), enabling virtual musical consumption in private environments without the need for in-person attendance at concerts while being able to repeat the auditions at will (Uribe, 2013) since the digital format allows digital musical storage (Wortman, 2019). The technological transformation of the uses and customs of a large part of the population, which has transitioned to the virtual from face-to-face, allows consumers to enjoy any music of their choice, a fact that had never occurred before (del De Moya Martínez et al., 2014).

As Wortman (2019) points out, digital platforms have brought to private domestic environments a large amount of musical content for personal enjoyment and its exchange with like-minded people or groups, generally through the use of social media. These have become a powerful element of social interaction as well as a way to share content of all types, especially among the youngest (Uribe, 2013). If a minimum of ethical personal commitment, respect, and responsibility is maintained, these digital activities are something positive that can help personal emotional growth and development (del De Moya Martínez et al., 2023).

Some authors (Tejada Garitano et al., 2019) point out that the current social relationship has been normalized digitally through the use of social media, i.e., sharing information and contacting other users, especially through TikTok and Instagram (Gozálvez-Pérez and Cortijo-Ruíz, 2023). Although these were already widely used, their use increased considerably during lockdown (Vázquez Chas, 2023), when the Internet, the network of networks, became the great ally of many isolated citizens who helped maintain human contact and alleviate their loneliness, anguish, and worry in the face of the serious situation caused by COVID-19.

Currently, social media is led by WhatsApp, Instagram, Facebook, and YouTube, with TikTok being the fastest-growing one (Redacción, 2023). A comparison with the year 2022 reveals that TikTok and Instagram have increased their users and that TikTok, Instagram, WhatsApp, and YouTube are preferred by consumers (IAB Spain, 2022, as cited in Redacción, 2023). It highlights the use of Twitter, Twitch, Tinder, Snapchat, and others as well (The 15 most used social media in 2023, 2023).

For years, the queen of social media was Facebook, with more than 2,900 million users worldwide (Galeano, 2023). However, currently, it has lost its appeal with the youth turning to Instagram (Vázquez Chas, 2023), among others, to follow *influencers* or content creators (Redacción, 2023). Young users have driven the growth of TikTok, where challenges, *trends,* music, and dance challenges prevail (Álvarez Álvarez, 2022). It is such a success that other applications also use short videos (Instagram *reels* or YouTube *shorts*). Spotify is a specific music consumption medium that has been growing since 2006 (Vela, 2020), marking a virtual milestone in the music industry with its *playlists* of all genres and musical styles. The popular YouTube platform hosts all types of content and allows us to listen to and discover music in its numerous hosted audiovisual materials, effectively satisfying its users (Agis et al., 2023).

## 2 Methodology

This study aims to know, analyze, and assess the incidence of music consumption through the use of technologies during lockdown. As secondary objectives, finding out the most used technological tools and establishing an assessment of musical habits in everyday life stand out. To respond to the purpose of the research, we performed a quantitative approach study with a descriptive design through a survey, which was supplemented with qualitative questions collected through the same questionnaire. Thus, the manner in which the diffusion, impact, and presence of music in everyday activities occur through the use of IT and social media was deepened, focusing on the Italian context during the COVID-19 pandemic.

The quantitative approach is used to collect data objectively for further analysis based on which different hypotheses are established and conclusions are drawn (Sampieri et al., 2003), while descriptive design, in this case by survey, can be defined as a scientific method that involves observing and describing the behavior of a subject without influencing it whatsoever (Shuttleworth, 2019). Conversely, regarding the qualitative approach, Álvarez-Gayou (2004) points out that this method regularly uses research questions and collects data with no numerical measurement. It is a flexible process that moves between the events that happen and the interpretation that is made of them. It reconstructs reality and is usually known as a “holistic” method based on inductive schemes, in which it is based on premises whose truth supports the conclusion but does not guarantee it.

The data collection was performed in the Italian Veneto area in 2022, during a research stay at the University of Padua, to know the musical habits of northern Italians during the pandemic to subsequently make comparisons with other studies performed in Spain.

The *ad hoc*-developed questionnaire was administered to a random probability sample through its dissemination through social media and email using the “snowball” procedure. In this type of sampling, the sample is selected from a larger population, including individuals chosen at random and by pure chance. In this regard, each individual has the same probability of being chosen at any stage of the process (Otzen and Manterola, 2017). A total of 338 responses comprised the population sample of the study (*N* = 338). The questionnaire consists of demographic (4 calibration questions), quantitative (X questions with answers following a Likert scale), and qualitative [3 > (X questions)].

To analyze these data and treat them, going through three phases during the part prior to the presentation of the results is crucial. Therefore, the methodology section is organized into three parts, corresponding to the steps that we followed to obtain the relevant results from the questionnaires. First, we focus on the preparation prior to the statistical analysis phase that has been performed through the Microsoft Excel^1^ software. We then explain the work performed with the aid of the SPSS software^2^ to specifically treat the exported quantitative data from this modified Excel in a second part. Finally, in the third section, we show the treatment of qualitative data that have been analyzed through another specific software, ATLAS.ti.^3^

### 2.1 Pre-coding

First, it was necessary to work with the Excel software to export the data and format it numerically.

In the file of responses to the questionnaire, many of the data appeared in nominal and numeral form simultaneously. There were also numerous variables in the form of scales and conditions. To facilitate the work, these data had to be encoded in a numerical format and thus exported to the SPSS 28 software. To simplify and modify the raw data in the Excel file, specific formulas, such as the SI encoding function, were used. The objective of this coding was to change this data to a numeral format, e.g., *woman = 1* and *man = 2*. Depending on the number of nominal variables to convert into the numeral format, the number of SI conditions increased; therefore, variables such as *“work” or “place of residence”* would have a coding of up to 7 or more. A decision to add a “*Did Not Answer”* variable in the unanswered cells was made. A number for this option was added to the coding formulas and the treatment of the other outcomes was facilitated. Some questions allowed multiple answers and had to be separated into several questions with dichotomous answers (e.g., “*musical genre”* or “*places where music is listened to*”). To allow its subsequent treatment, coding was performed by adding nine columns for each possible answer and leaving the option of *yes*/*no/no answer.* The question about the musical genre left an option for 11 different answers that have become ten independent questions with dichotomous answers. The question “*What is the main musical genre you listen to?*” became “*Do you listen to classical music? Jazz music?* …” and so on for the ten possibilities proposed in the questionnaire with the idea of handling only numerical data and facilitating its export to SPSS 28.

The same coding process was used for questions specifying a *measurement intensity/scale* in the use of platforms or devices. Another necessary coding has been to propose ranges or groups for the variables of sports time or frequency per week.

### 2.2 SPSS coding

Once all the previous work, changing the nominal variables into numerical codes was performed using Excel, the data were analyzed through the SPSS software. The results were imported numerically, and the different variables were coded according to the changes in the Excel software. Three types of information had to be processed and codified: nominal data that allowed only one answer to be selected according to the range in which the participant is or according to the answer that the person has to propose.

In these types of questions, numbers were proposed for each different answer, e.g., information about age, city, activity performed, and others. Similarly, the questions that allow us to determine if the consumption of music has increased, decreased, or remained the same are the level of musical training, spending on music, and others. These answers also have to be coded based on numbers to be treated statistically. Then, we had to deal with questions of scales that were conditioned with a previous dichotomous question in which the person had to say whether they used a platform/device or not, and if so, place their use from 1 to 5. There have been several of these questions, and double-coding was required. First, we separated the questions related to use individually, coding as follows: 1 = “*Yes,” 2 = “No”*. We then used the SPSS software to “set aside” the questions corresponding to the number and treat this data without having lost cases. Thus, the analysis was more accurate and in line with the actual data. This operation was performed again for each of these questions (*device, platform,* etc.). When coding the questions with multiple answers that allow us to have several answers within those that are proposed, we used the questions already modified in Excel and coded them within the SPSS software so that they stayed with answers in the form of 1 = “*Yes,” 2 = “No,”* and *3 = “DNA”*. This coding provides results that correspond to the frequencies of each category but does not give us the option to analyze if a person has selected several options. In this case, we have to make cross-tables to check the correlation between different variables. Notably, here, we find a lot of information, but independently.

The use of the SPSS software allows us to have a graphical view and tables of the different variables once the descriptive analysis of each piece of information is performed. Depending on the data we are going to deal with, it is better to have pie or histogram graphs to compare the data. Previously, we worked with the tables that collect the data before we could analyze them visually. SPSS is that it can add the data within the graphs and thus directly see the frequencies or percentages within the corresponding place in the graphic representations (Castañeda et al., 2010).

### 2.3 Qualitative data

Open-ended questions with no choice from a list of answers were treated as qualitative data. Therefore, we used a third software, ATLAS.ti, which allows the analysis of this type of data. Through this software, we have treated some variables, such as the specialties that those respondents who are enrolled in university studies are studying. We also have the platforms or social media that were used during the lockdown, since the people who have been questioned could express themselves freely. To conclude the questionnaire, we managed two other open-ended questions. The first was to allow the people questioned to express their preferences for the artists or musical groups they usually listen to. Therefore, the answers are open and without restrictions, which allows one to obtain numerous different answers. Similarly, to finish the questionnaire, it has been proposed to define music with some words, which leaves room for numerous answers. Therefore, we treated these data qualitatively because the people questioned had answered with single words, but some also composed their answers in paragraphs. The software allows us to process these data as frequency and word clouds to graphically visualize the different results. Therefore, answers that are repeated more times will appear with a larger size and more striking colors than the ones that usually come out once. To use this data, it is necessary to export the Excel columns to a Word document to delete all the useless words that enter the data processing. Therefore, we deleted articles and connectors such as “*and*” and “*of*” so that these are not counted. When the answer is more than one word, to prevent the software from recognizing it as different data, the words are joined with an underscore “_” and thus appear as a single answer.

The answers to the last question related to the words representative of the music and that were in paragraph format have been treated separately to avoid an encoding that would have been more complex and that would have complicated the treatment of the data. Once these three steps were completed, the following results, which are described in detail in the next section, were obtained.

## 3 Results and discussion

In this section, we analyze the results according to the main objective of the study (to know, analyze, and assess the incidence of music consumption through the use of technologies during lockdown) and the secondary objectives (to identify the most used technological tools and assess musical habits in everyday life).

The analysis of the results follows three steps. First, the results of the calibration responses are described. Subsequently, the quantitative responses are analyzed. Finally, the qualitative results are discussed. For a better understanding, all results are presented in the tables and graphs found in the annexes.

### 3.1 Calibration responses

These questions establish the sample profile to determine whether there is a relationship between it and the results. For age (*Cf.* Supplementary Table S1 and Graph 1 in Supplementary material), there are two clear groups: the youngest and the oldest are the most numerous, and there is less presence of the intermediate age. The most represented age range corresponds to the age between 55 and 64 years with 26.33% (89 people), followed by 18–24 years with 23.37%. The intermediate ages (25–34 years, 35–44 years, and 45–54 years) have a representation of 10%.

Larger age groups can have an impact on results because they involve different rhythms of life (students, professionals, and retirees).

As for sex (*Cf.* Supplementary Table S2) there is more female than male participation. It would be interesting to verify whether gender has an influence on the musical habits. Among the 338 people surveyed, two did not answer, and the remainder are divided into 42.6% of men (144 people) and 56.8% of women (192). Additionally, the possible relationship between age and sex of the sample was verified (*Cf.* Supplementary Table S3) and the relationship between the two variables and female predominance in the two most representative age groups was evidenced with some parity in the remainder. Therefore, the relationship between sex and the most representative ages is verified since the majority of the sample is composed of young and older women. In the socio-professional category (*Cf.* Supplementary Table S4), three groups are delimited: students, employees/workers, and those without professional activity (unemployed or retired). It is evident (*Cf.* Graphs 5, 6 in Supplementary material) that employees/workers are the largest group, with 51.2% (173 people), followed by first and second cycle students (21.3 and 3.6%, respectively), master’s degrees (1.2%), and doctorates (1.5%). The last category brings together retirees (59) and the unemployed (11) at 20.8%.

Regarding the population of Veneto, the majority of the sample (63.6%) came from Padua with 215 responses (*Cf.* Supplementary Table S6), while the other areas are few significant and 22 people did not answer. Other cities are Venice (31 people, 9.2%), Belluno (5 people, 1.5%), Verona (9 people, 2.7%), Rovigo (2 people, 0.6%), Treviso (22 people), and Vicenza (32 people, i.e., 9.5%).

### 3.2 Quantitative responses

#### 3.2.1 Use of devices and platforms

##### 3.2.1.1 Devices

The data collected gives an idea of the different devices and platforms that respondents usually use. Through the tables found in the annexes, it is analyzed whether or not certain types of devices are used (*Cf.* Table 1) and the intensity of use of each device (*Cf.* Table 2). The devices are those usually used in everyday life: computers, tablets, iPads, radios, televisions, and mobile phones.

**Table 1:** Use of devices.

**Q5:** Regarding the use of technologies, which are the devices you use the most?					
		Frequency	Percentage	Valid percentage	Cumulative percentage
Computer	Yes	327	96,7	96,7	96,7
	No	11	3,3	3,3	100,0
	Total	338	100,0	100,0	
Tablet	Yes	279	82,5	82,5	82,5
	No	59	17,5	17,5	100,0
	Total	338	100,0	100,0	
iPad	Yes	272	80,5	80,5	80,5
	No	66	19,5	19,5	100,0
	Total	338	100,0	100,0	
Television	Yes	325	96,2	96,2	96,2
	No	13	3,8	3,8	100,0
	Total	338	100,0	100,0	
Radio	Yes	317	93,8	93,8	93,8
	No	21	6,2	6,2	100,0
	Total	338	100,0	100,0	
Cellulare	Yes	331	97,9	97,9	97,9
	No	7	2,1	2,1	100,0
	Total	338	100,0	100,0	

**Table 2:** Assessment of computer usage.

**Q5.1:** Rate your use from 1 to 5, with 1 being the lowest and 5 being the highest.					
		Frequency	Percentage	Valid percentage	Cumulative percentage
Computer	1	28	8,6	8,6	8,6
	2	17	5,2	5,2	13,8
	3	56	17,1	17,1	30,9
	4	89	27,2	27,2	58,1
	5	137	41,9	41,9	100,0
	Total	327	100,0	100,0	
Tablet	1	181	64,9	64,9	64,9
	2	38	13,6	13,6	78,5
	3	25	9,0	9,0	87,5
	4	21	7,5	7,5	95,0
	5	14	5,0	5,0	100,0
	Total	279	100,0	100,0	
iPad	1	184	67,6	67,6	67,6
	2	25	9,2	9,2	76,8
	3	20	7,4	7,4	84,2
	4	18	6,6	6,6	90,8
	5	25	9,2	9,2	100,0
	Total	272	100,0	100,0	
Television	1	65	20,0	20,0	20,0
	2	63	19,4	19,4	39,4
	3	99	30,5	30,5	69,8
	4	61	18,8	18,8	88,6
	5	37	11,4	11,4	100,0
	Total	325	100,0	100,0	
Radio	1	99	31,2	31,2	31,2
	2	69	21,8	21,8	53,0
	3	72	22,7	22,7	75,7
	4	37	11,7	11,7	87,4
	5	40	12,6	12,6	100,0
	Total	317	100,0	100,0	
Cellulare	1	5	1,5	1,5	1,5
	2	6	1,8	1,8	3,3
	3	24	7,3	7,3	10,6
	4	64	19,3	19,3	29,9
	5	232	70,1	70,1	100,0
	Total	331	100,0	100,0	

The computer is used by 96.7% of the sample, compared to 3.3% who do not use it; tablets, 82.5%, compared to 17.5% who do not use them. There are 272 people who use iPads, with this use and its assessment being directly related to the overall use of the tablet. TV is present in all homes and is one of the most used (325 people compared to 13 who do not use it regularly). The use of IT would suggest the disappearance of radio, but 93.8% use it. And the star technological product is the mobile phone (used by 97.9%).

In the assessment of the use of these devices, the computer is well considered: 89 people rate it at 4 (27.2%) and 137 people at 5 (41.9%), while 1, 2, and 3 are well below. Tablet has a low rating: 181 people (64.9%) grant 1 versus 5 (5%), and very low 1, 2, 3 (15%). iPad users rate it low (67.6%), and the remainder of the ratings have lower percentages that do not exceed 10% for each. TV use is distributed among the 5 ratings, with the most represented being the intermediate (30.5%) compared to the rest (around 20%). For radio, the most represented rating is the lowest (31.2%). Mobile phone is very high (232 people (70.1%) rate it at 5), not significant, and the lowest (less than 2%). This corroborates that technology collaborates with listening to achieve the musical experience (Warner, 2017).

##### 3.2.1.2 Platforms

Platforms and assessments of their use (*Cf.* Tables 3, 4) state that 10 platforms are detected, most of them known, but some are new or unknown. Generally, the use is homogeneous, and each platform has more than 70% use. A total of 329 people use WhatsApp (97.3%), 287 use Instagram (84.9%), 317 use Youtube (93.8%), 263 opt for PodCast (77.8%), 294 are on Facebook (87%), 253 use SoundCloud (74.9%), and 262 use Telegram (77.5%). Spotify, BandCamp, and iTunes are used by 271, 247, and 250 people, respectively (80.2, 73.1, and 74%). The most used is WhatsApp, and the least is BandCamp. The assessment of use is heterogeneous: WhatsApp is the most used and well rated (65% maximum rating); the rest are less valued, although they are among the most used; Facebook and Youtube have lower ratings.

**Table 3:** Use of the different platforms.

**Q6.1:** Which platforms do you use the most?					
		Frequency	Percentage	Valid percentage	Cumulative percentage
WhatsApp	Yes	329	97,3	97,3	97,3
	No	9	2,7	2,7	100,0
	Total	338	100,0	100,0	
Instagram	Yes	287	84,9	84,9	84,9
	No	51	15,1	15,1	100,0
	Total	338	100,0	100,0	
YouTube	Yes	317	93,8	93,8	93,8
	No	21	6,2	6,2	100,0
	Total	338	100,0	100,0	
PodCast	Yes	263	77,8	77,8	77,8
	No	75	22,2	22,2	100,0
	Total	338	100,0	100,0	
FaceBook	Yes	294	87,0	87,0	87,0
	No	44	13,0	13,0	100,0
	Total	338	100,0	100,0	
SoundCloud	Yes	253	74,9	74,9	74,9
	No	85	25,1	25,1	100,0
	Total	338	100,0	100,0	
Telegram	Yes	262	77,5	77,5	77,5
	No	76	22,5	22,5	100,0
	Total	338	100,0	100,0	
Spotify	Yes	271	80,2	80,2	80,2
	No	67	19,8	19,8	100,0
	Total	338	100,0	100,0	
BandCamp	Yes	247	73,1	73,1	73,1
	No	91	26,9	26,9	100,0
	Total	338	100,0	100,0	
iTunes	Yes	250	74,0	74,0	74,0
	No	88	26,0	26,0	100,0
	Total	338	100,0	100,0	

**Table 4:** Assessment of the use of the platforms.

**Q6.1:** Rate your use from 1 to 5.					
		Frequency	Percentage	Valid percentage	Cumulative percentage
WhatsApp	1	5	1,5	1,5	1,5
	2	9	2,7	2,7	4,3
	3	26	7,9	7,9	12,2
	4	75	22,8	22,8	35,0
	5	214	65,0	65,0	100,0
	Total	329	100,0	100,0	
Instagram	1	91	31,7	31,7	31,7
	2	34	11,8	11,8	43,6
	3	42	14,6	14,6	58,2
	4	46	16,0	16,0	74,2
	5	74	25,8	25,8	100,0
	Total	287	100,0	100,0	
YouTube	1	31	9,8	9,8	9,8
	2	56	17,7	17,7	27,4
	3	78	24,6	24,6	52,1
	4	81	25,6	25,6	77,6
	5	71	22,4	22,4	100,0
	Total	317	100,0	100,0	
PodCast	1	157	59,7	59,7	59,7
	2	42	16,0	16,0	75,7
	3	34	12,9	12,9	88,6
	4	16	6,1	6,1	94,7
	5	14	5,3	5,3	100,0
	Total	263	100,0	100,0	
FaceBook	1	98	33,3	33,3	33,3
	2	37	12,6	12,6	45,9
	3	56	19,0	19,0	65,0
	4	47	16,0	16,0	81,0
	5	56	19,0	19,0	100,0
	Total	294	100,0	100,0	
SoundCloud	1	212	83,8	83,8	83,8
	2	15	5,9	5,9	89,7
	3	15	5,9	5,9	95,7
	4	7	2,8	2,8	98,4
	5	4	1,6	1,6	100,0
	Total	253	100,0	100,0	
Telegram	1	146	55,7	55,7	55,7
	2	54	20,6	20,6	76,3
	3	33	12,6	12,6	88,9
	4	17	6,5	6,5	95,4
	5	12	4,6	4,6	100,0
	Total	262	100,0	100,0	
Spotify	1	112	41,3	41,3	41,3
	2	32	11,8	11,8	53,1
	3	32	11,8	11,8	64,9
	4	32	11,8	11,8	76,8
	5	63	23,2	23,2	100,0
	Total	271	100,0	100,0	
BandCamp	1	230	93,1	93,1	93,1
	2	5	2,0	2,0	95,1
	3	3	1,2	1,2	96,4
	4	5	2,0	2,0	98,4
	5	4	1,6	1,6	100,0
	Total	247	100,0	100,0	
iTunes	1	208	83,2	83,2	83,2
	2	13	5,2	5,2	88,4
	3	12	4,8	4,8	93,2
	4	5	2,0	2,0	95,2
	5	12	4,8	4,8	100,0
	Total	250	100,0	100,0	

#### 3.2.2 Music and everyday life

Observing the relationship between listening to music and everyday life (*Cf.* Tables 5, 6), it is evident that the majority of the sample listens to music. Among the 338 people, 326 have musical habits in their everyday lives (96.4%). Only 11 people answered negatively (3.3%), and 1 did not answer. Regarding the place and context of listening (*Cf.* Table 6), the majority of answers are *for pleasure* (75.2%), *in the car* (69.3%), and *during relaxation moments* (70.6%). The least frequent answers are *while doing homework* (9.8%) or *for work purposes* (11.3%). Intermediate percentages are *at the workplace*, *on the street*, *while playing sports,* or *walking* (between 38 and 24.8%) (see Table 7).

**Table 5:** Do you listen to music in your everyday life?

**Q7:** Do you listen to music in your everyday life?					
		Frequency	Percentage	Valid percentage	Cumulative percentage
	Yes	326	96,4	96,4	96,4
	No	11	3,3	3,3	99.7
	DNA	1	0.3	0.3	100,0
	Total	338	100,0	100,0	

**Table 6:** Place where music was listened to.

**Q7:** Indicate where did you listen to music					
		Frequency	Percentage	Valid percentage	Cumulative percentage
At the workplace	Yes	81	24.8	24.8	24.8
	No	245	75.2	75.2	100,0
	Total	326	100,0	100,0	
In the car	Yes	226	69.3	69.3	69.3
	No	100	30.7	30.7	100,0
	Total	326	100,0	100,0	
During relaxation moments	Yes	230	70.6	70.6	70.6
	No	96	29.4	29.4	100,0
	Total	326	100,0	100,0	
While doing homework	Yes	32	9,8	9,8	9,8
	No	294	90.2	90.2	100,0
	Total	326	100,0	100,0	
While doing sports	Yes	96	29.4	29.4	29.4
	No	230	70.6	70.6	100,0
	Total	326	100,0	100,0	
While walking on the street	Yes	112	34.4	34.4	34.4
	No	214	65.6	65.6	100,0
	Total	326	100,0	100,0	
During a concert	Yes	124	38.0	38.0	38.0
	No	202	62.0	62.0	100,0
	Total	326	100,0	100,0	
For pleasure	Yes	245	75.2	75.2	75.2
	No	81	24.8	24.8	100,0
	Total	326	100,0	100,0	
For work purposes	Yes	37	11.3	11.3	11.3
	No	289	88.7	88.7	100,0
	Total	326	100,0	100,0	

**Table 7:** Changes during lockdown.

**Q8:** How did your listening to music change during the lockdown?				
	Frequency	Percentage	Valid percentage	Cumulative percentage
Increased	144	42.6	42.6	42.6
Remained the same	175	51.8	51.8	94.4
Decreased	17	5,0	5,0	99.4
DNA	2	0.6	0.6	100,0
Total	338	100,0	100,0	

#### 3.2.3 Changes during the pandemic

Changes in musical habits during the lockdown are noted (*Cf.* Supplementary Table S13; Supplementary Figure S9). Before the pandemic, there were no changes (51.8%, 144 people), while 42.6% stated that they listened to music more, and few people realized their musical habits had been reduced.

Variations (*a little, quite a bit,* or *a lot*) in listening to music (*cf.* Supplementary Table S14; Supplementary Figure S10) show that listening to music has changed little for the group (63.9%), although others consider that it has changed a lot (9.5%) or quite a bit (24.6%).

Changes in music listening habits during the pandemic (*cf.* Supplementary Tables S15, S16; Supplementary Figure S11) of people surveyed, on a scale from 1 (worst) to 5 (best), show that 119 people (40%) observed a change. The assessment of this change consisted of *enjoying music* and *appreciating listening more* (*Cf.* Supplementary Table S17; Supplementary Figure S12) with the election of 4 (26.1%), followed by 5 (14.3%) and 3 (12.6%). 45 people did not answer (37.8%). Assessments of 1 and 2 offer non-significant percentages.

Three changes are observed in the following musical habits: *discovering new performers* (*cf.* Supplementary Table S18; Supplementary Figure S13), and its assessment shows an average/high average of 19.3% (3) and 14.3% (4) compared to 18.5% (22 people chose 5). There were few negative ratings, and 40 people did not answer. For *discovering a new music genre* (*cf.* Supplementary Table S19; Supplementary Figure S14), the most chosen option was 3 (23 answers, 19.3%). The lowest ratings (1 and 2) are 18.5% and the highest are 25, and 36.1% did not answer (43 people). As for *discovering a new musical genre* (*cf.* Supplementary Table S19; Supplementary Figure S14), rating 3 stands out (23 people, 19.3%). Meanwhile, 1 and 2 account for 18.5%, and 4 and 5 account for 25%. Forty-three people did not answer (36.1%). Finally, as for *relevance of small things such as listening to music* (*cf.* Supplementary Table S20; Supplementary Figure S15),19.3% chose 5, 20.2% chose 4, and 15.1% chose 3, with 1 and 2 accounting for less than 10%.

Changes in musical habits are positive and well valued by people surveyed, with the main reasons being: *I like it* (79.3%), *it is important in my everyday life* (51.2%), *it entertains me* (40.2%), *it boosts my morale* (37.9%), *it keeps me company* (34.3%), *I’m used to it* (17.8%), “*did not answer*” (*cf.* Supplementary Table S21). Other reasons or lack of answers are not significant, with percentages close to 0.

The majority of respondents do not share audiovisual or musical material on social media (253 people, 74.9%) compared to those who do [72 (21.3%)]. Difficulties encountered during lockdown stand out (*cf.* Supplementary Table S23; Supplementary Figure S17), with *isolation from family or friends* (121 people, 35.8%) being the most chosen option, followed by *fear or insecurity* (73, 21.6%) or *boredom* (55, 16.3%). Other types of difficulties, such as *financial issues*, were specified in “*Others*” (18 people).

Among the answers about the help provided by music (*cf.* Supplementary Table S24), *improving mood* (213 people, 63%) stands out, followed by other responses: *satisfaction* (23.1%); *rest* (21%); *help at the workplace* (13.9%); *to relate to another person* (10.9%).

Four groups are observed for the music followed on social media (*cf.* Supplementary Table S25). The most representative is the *classic or usual theme* (157 people, 46.4%), followed by the *preferred theme* (102, 30.2%), *message of hope* (74, 21.9%), *new releases*, and those that did not answer (less than 15%).

Regarding the assessment of music relevance (*cf.* Supplementary Table S26; Supplementary Figure S18), the most representative score is 5 (28.7%), followed by 3 (27.5%) and 4 (27.2%), while 1 and 2 do not exceed 11%.

The last appreciation experienced during the pandemic was viewing or listening to music performances (*cf.* Supplementary Table S27; Supplementary Figure S19), either live or digital, and listening through technological means. This offers a balance between those who attended musical performances (139 people) and those who did not (192, 56.8%).

#### 3.2.4 Music and physical activity

The correlation between music and physical activity shows 221 people (65.4%) who exercise themselves (*cf.* Supplementary Table S28; Supplementary Figure S20) compared to 32.8% who do not, with 6 not answering (not significant). As for the frequency of weekly (*cf.* Supplementary Table S29; Supplementary Figure S21) or daily sports activity (*cf.* Supplementary Table S30; Supplementary Figure S22), the majority of respondents chose the “*3 times or less*” option: *once* (5.6%), t*wo or three days* (18.6 and 21.9%, respectively). Frequencies greater than 3 days per week obtained lower percentages and, thus, are not considered relevant. As for the union of physical exercise and listening to music (*cf.* Supplementary Table S31; Supplementary Figure S23), 146 people answered “Yes” (43.2%); “No” (38.8%); and 18% did not answer.

#### 3.2.5 Listening to music

Weekly listening of the sample (*cf.* Supplementary Table S32; Supplementary Figure S24) yielded the following data: The most frequent option is listening to music “*every day*” (229 people, 67.8%); “*5 times a week*” (10.9%, 37 people); “*3 times a week*” (14.2%, 48 people); “*less than once a week*” (5.3%); and “*did not answer*” (1.8%).

Monthly expenditures on music during the pandemic (*cf.* Supplementary Table S33; Supplementary Figure S25) showed that the majority of respondents avoided or limited such expenses. 229 people did not spend money on music during the pandemic (67.8%), and 66 spent a mean of €10 per month (19.5%). Those who spent the most, €20, comprise less than 15% (21 people), and 17 people spent more than €20.

Regarding the musical style listened to during the pandemic (*cf.* Supplementary Table S34), the most listened-to style was pop music (60.7%), followed by *rock, classical music*, and *jazz*, while *dance*, *electronic*, or *popular* music were not listened to at all. Category *“Otros”* is common, although it has not been possible to verify what style of music the respondents were referring to.

Regarding music training (*cf.* Supplementary Table S35; Supplementary Figure S26), 134 people (39.6%) have no training; little, 31.1%; intermediate, 18.3%; and a minority group has a high level of musical training.

### 3.3 Qualitative responses

The analysis of some questions could not be assessed quantitatively as it was not possible to numerically code the answers; therefore, we resorted to analyzing and showing the results visually, as word clouds.

**Figure 1:**
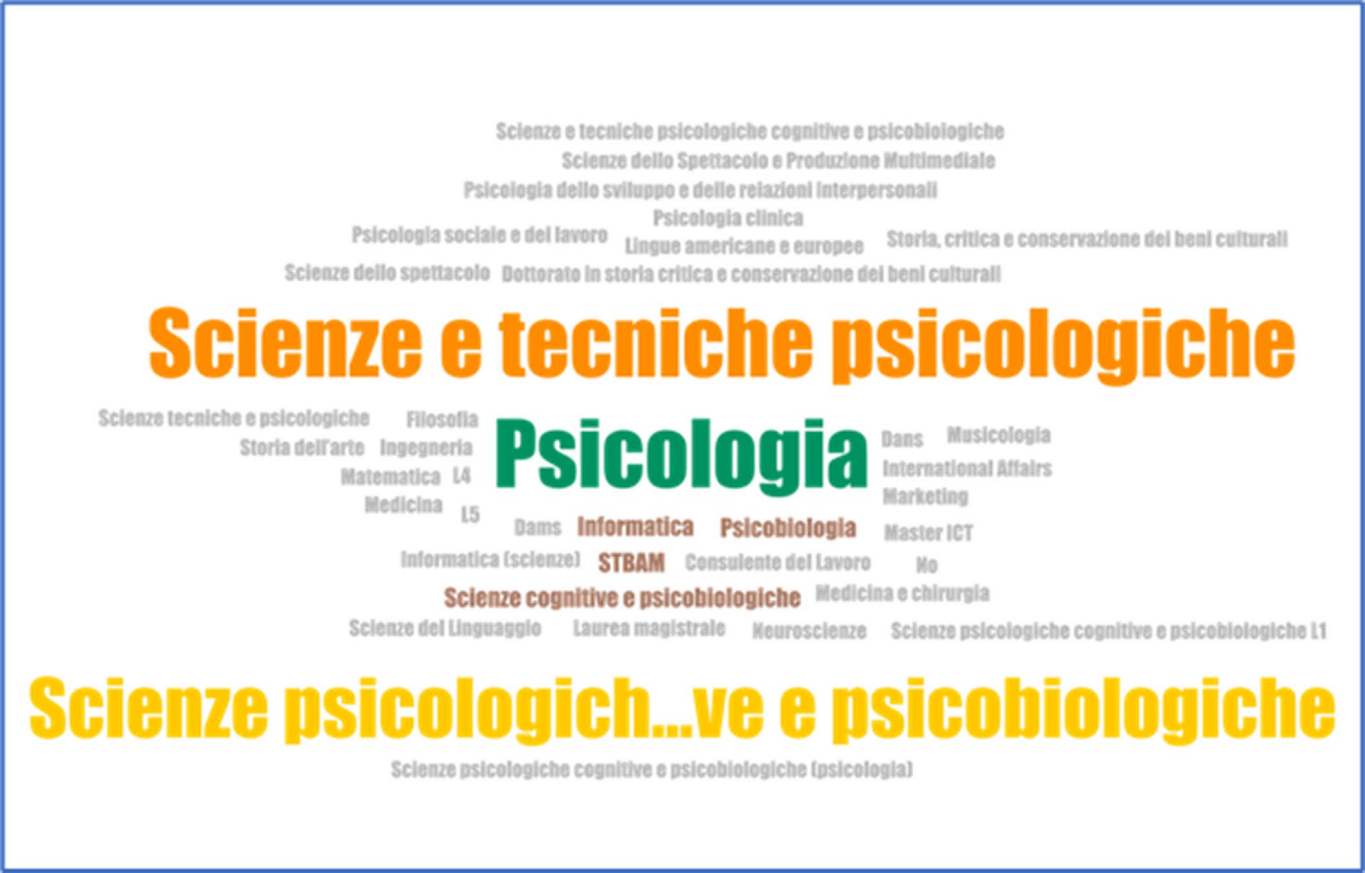
Most frequent specialties.

The question “*If you are a university student, indicate your specialty or field of study*” (coding Q3_1) aims to know precisely the studies of university students. Using the ATLAS.ti software, a word cloud showing the most frequent specialties was generated (*cf.* Figure 1).

The most abundant studies appear in color, careers related to *Psychology* (psychology, psychobiology, and psychological sciences and techniques), and in gray, those that were only chosen once.

**Figure 2:**
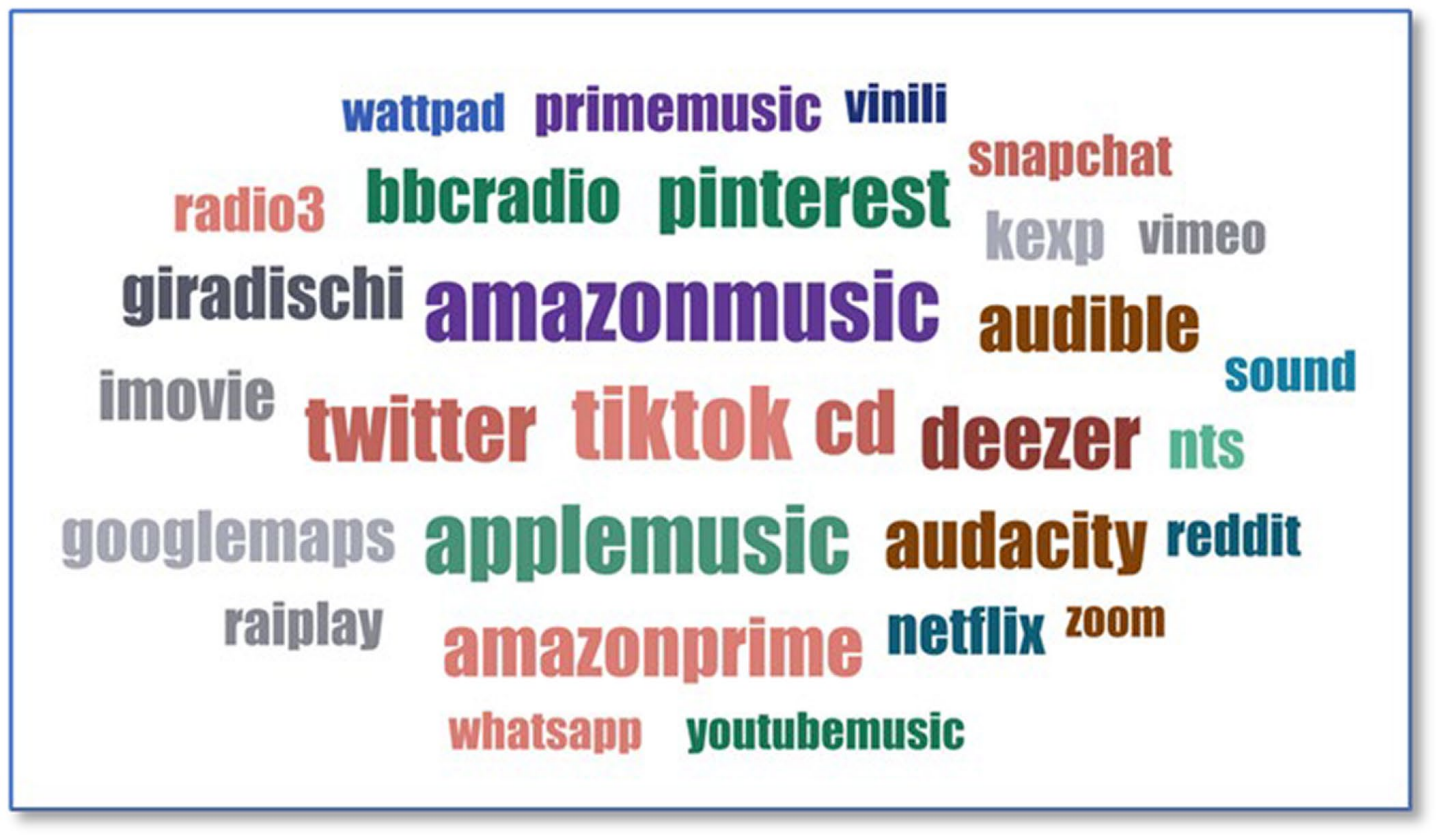
Most used platform.

The question “*Which platform do you use the most*?” is the last of a series of 11 dichotomous questions that allow data not foreseen in the response possibilities to be collected. The answers review platforms that are not associated with exclusive music listening (*cf.* Figure 2).

For music consumption, some are Italian, such as *Raiplay* or *radio3*. The most used are paid apps or social media, such as *TikTok*, *Amazon Music,* or *Apple Music*. Other media, such as CDs and vinyl, are observed as well. Most of these platforms are well-known, and not all of them are exclusively music-oriented, since some of them, such as *Netflix*, *YouTube,* or *Vimeo,* are streaming platforms.

**Figure 3:**
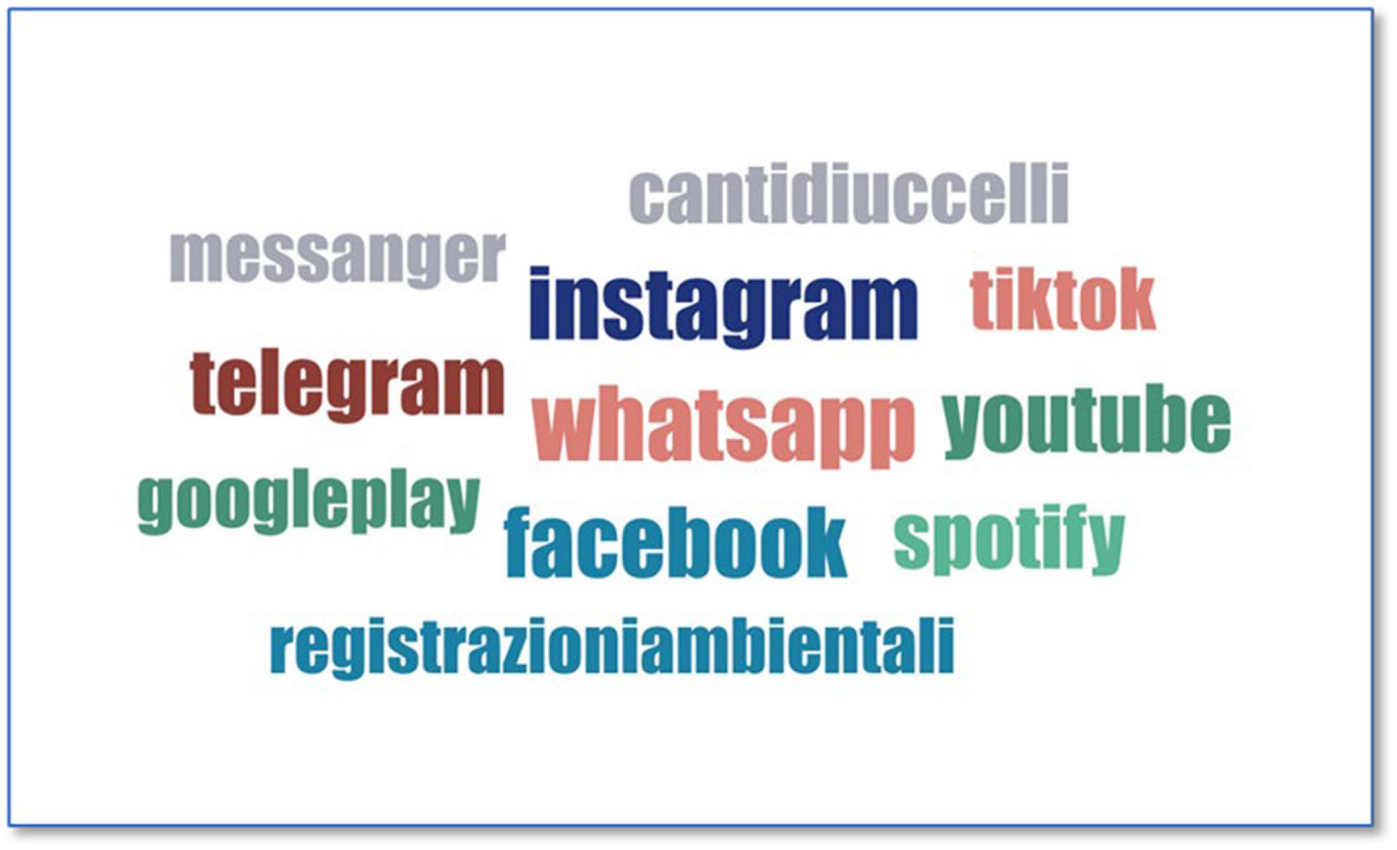
Social media to share recordings.

For the question “*Did you share your musical recordings on social media during the lockdown*?” the social media where such recordings were usually published or shared had to be specified, allowing the possibility of writing any type of information, in this case, social media (“*If yes, please indicate on which social media did you share the recordings*”). The responses were grouped, and the data were processed using the ATLAS.ti software, from which the following word cloud was obtained (*cf.* Figure 3).

Repeating words are highlighted in gray. Most of them are the main social media platforms found across the Internet, interactive messaging platforms where audiovisual material such as videos or photos can be shared. These platforms developed more during lockdown, possibly because these are windows to the outside. The most used social media is WhatsApp, located in the center and surrounded by others. There are apps or social media platforms such as TikTok, Facebook, and Instagram. There is also Telegram, which is similar to WhatsApp, albeit not in a central location. Spotify is specifically musical. Other apps are local/national, such as “Cantidiuccelli”. Therefore, the responses are not limited to international or famous social media.

**Figure 4:**
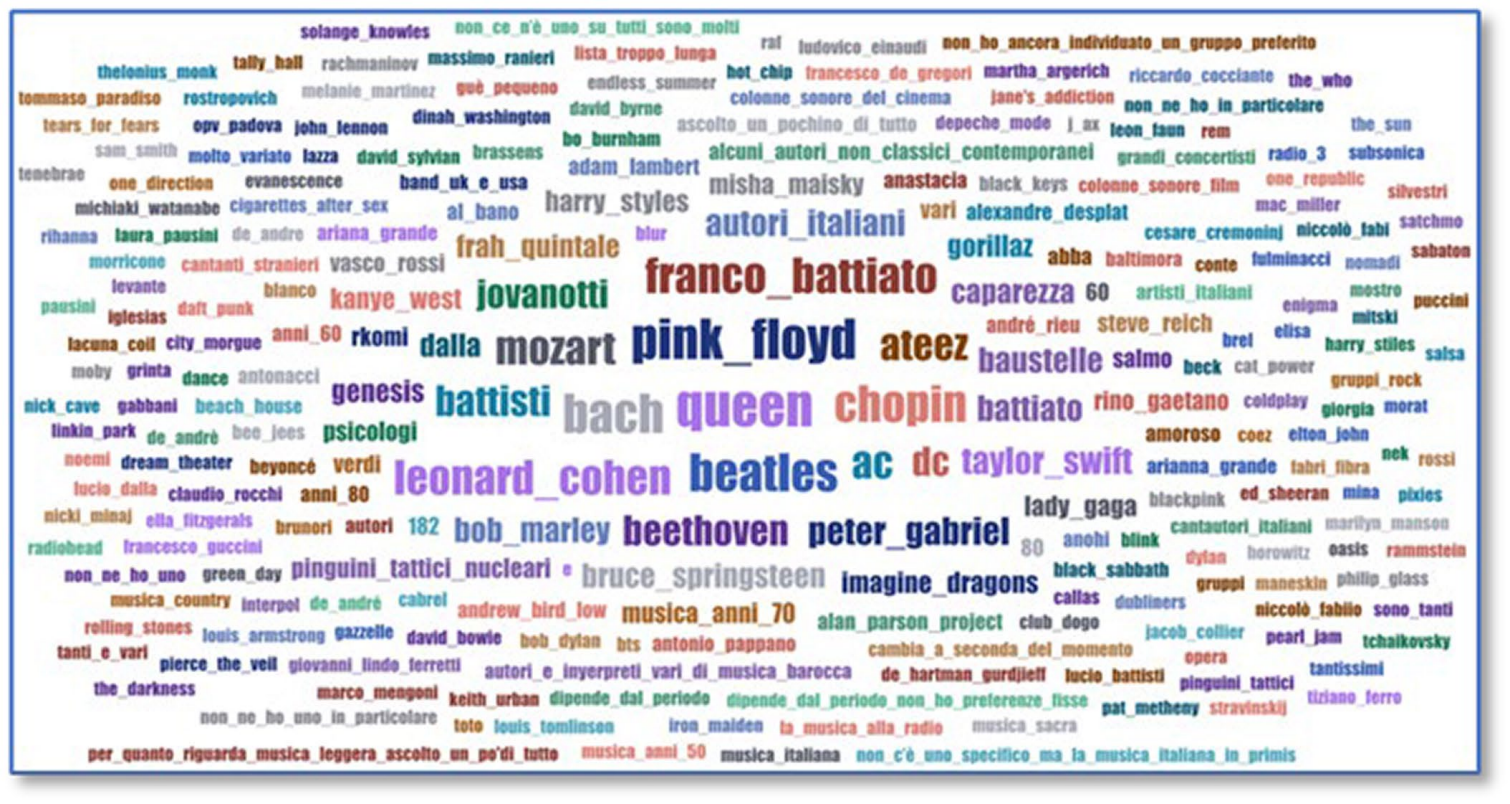
Favorite author/group/performer.

There are two key questions. The first one (*Who is your favorite author/group/performer*?) collects data on groups, authors, or performers that were listened to without restrictions on numbers or origin. As no limits were set in the responses, everyone expressed themselves freely; some chose several performers or groups, and others specified only one. The results show a wide variety of responses, although the most repeated are from well-known and international groups/artists (*cf.* Figure 4).

**Figure 5:**
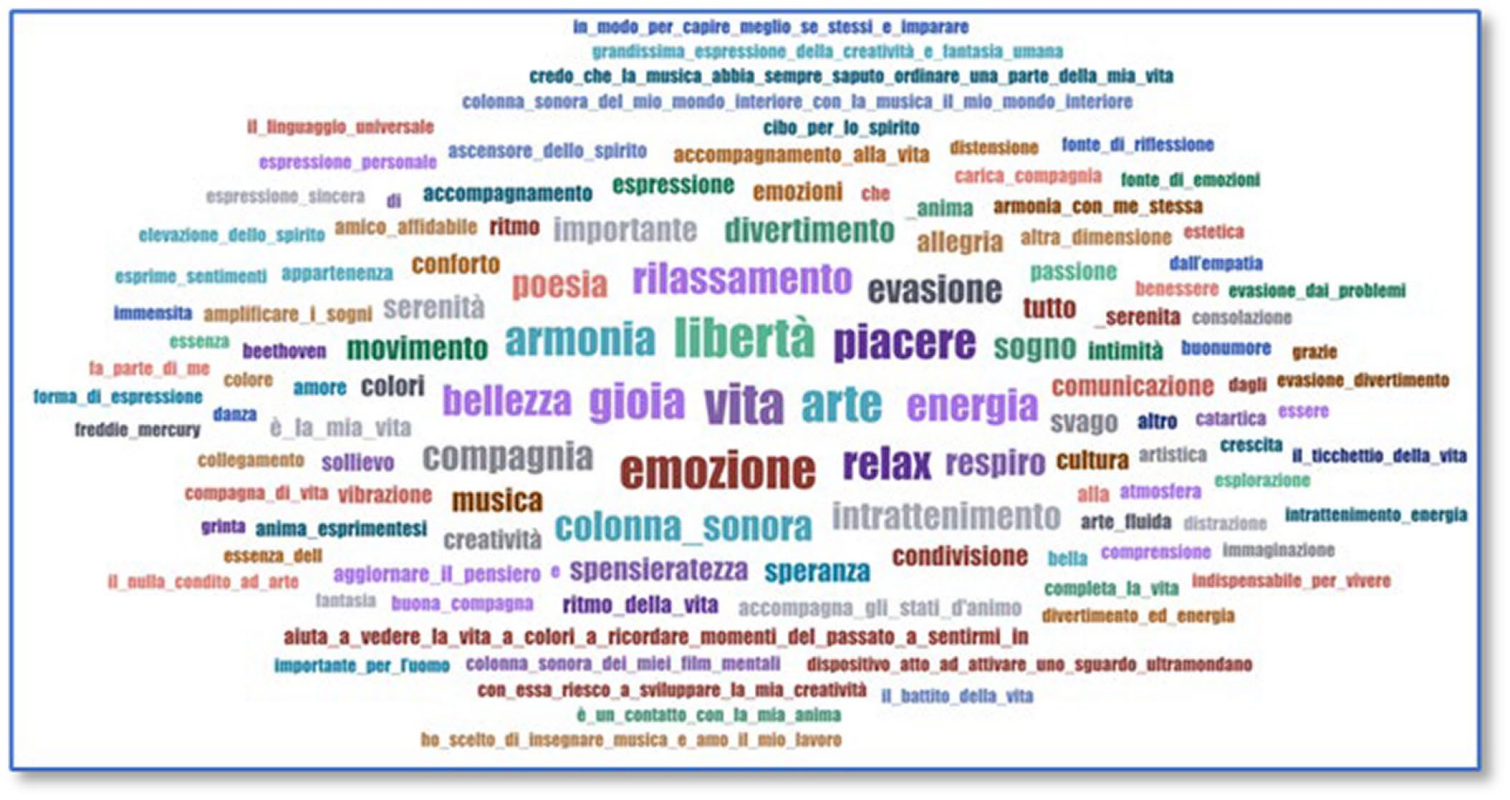
Music meaning.

The second question (*Indicate in one or two words what music means to you*) is aimed at knowing the listener’s emotions regarding music and the vital meaning that it has for the sample (*cf.* Figure 5).

## 4 Conclusion

The impact of the Internet and technologies on all areas of human beings’ everyday lives, personal experiences, and social relationships forces new key questions to emerge in this digital society. This study arises in this context. The interest of this study, focused on this recent period and of immense historical significance, must be sought in the role that technologies have played in the information and communication of people who were forced to stop physical contact and quickly adapt to a virtual one. The novelty of the topic and the sociological, cultural, and digital transformation implications have been a major challenge whose consequences are still being analyzed. In the musical field, it has had an impact on the way music is accessed and consumed through IT, which is why this research has also sought to determine the most used technological tools and an assessment of the musical habits in the everyday lives of the study participants.

The selection method for respondents, following random probabilistic sampling, has yielded that the age groups chosen at random were mainly between adults (55 to 64 years) and young people (18 to 24 years), followed by intermediate ages (between 25 and 54 years) and a practically equal distribution between genders, with the frequency of women being slightly higher. All of this offers a broad idea of the phenomenon that is intended to be described and allows for the establishment of a defined approach to the implications derived from it. This way, how electronic devices (computers, tablets, iPads, radios, televisions, and mobile phones) are widely used in the everyday lives of surveyed participants has been assessed. This fact undoubtedly facilitated access to the digital world during lockdown times, and in particular, to musical content. Among all of them, computers and mobile phones have emerged as the most popular means of accessing information and communication.

Regarding digital platforms and their more musical facet, the study found that the most used, in descending order, were WhatsApp, YouTube, Instagram, Facebook, Spotify, and podcast repositories. However, research has undoubtedly shown that music has had a prominent function during lockdown, being an activity performed out of pleasure whether at home, at work, or when walking alone. This reality has revealed the social and cultural function of music in people’s everyday lives. In fact, during the pandemic, the use and consumption of music have increased, making it a perfect ally to accompany people’s everyday lives thanks to its value in making people enjoy and discover new artists and musical genres thanks to the ease of access to other music provided by IT. The fact that music transmitted a message of hope and helped overcome moments of loneliness has drawn our attention.

Similarly, it has been observed how music has been present in people’s everyday lives, although according to the responses of surveyed participants, spending on listening to music was reduced during the pandemic, something that can be attributed to the situation of economic uncertainty while looking for alternatives such as online platforms that offered free music in exchange for advertising or resorting to existing recordings in homes. Regarding the musical style, the most listened to have been pop, rock, and jazz, followed by other more current music such as urban, dance, and electronic style, which allows us to venture the vital need to feel happy or entertained in the face of the dire reality experienced outside home at that time.

The prominence that social media have today, as well as the fascination they exert on the consumer, are irrefutable, and such topics are highly debated due to the lights and shadows they contain.

Overall, this study highlighted the power that music continues to exert in people’s everyday lives in a world dominated by technology. Although these tools have facilitated access and online musical consumption in the isolation period imposed by the pandemic, it has also been shown that the validity and relevance of the valuable material with which the music is constructed, rather than the medium, tool, or device, is what matters the most.

## Data availability statement

The original contributions presented in the study are included in the article/Supplementary material, further inquiries can be directed to the corresponding author.

## Ethics statement

Ethical approval was not required for the study involving humans in accordance with the local legislation and institutional requirements. Written informed consent to participate in this study was not required from the participants or the participants’ legal guardians/next of kin in accordance with the national legislation and the institutional requirements.

## Author contributions

MD: Writing – original draft, Writing – review & editing, Methodology, Supervision, Conceptualizationm, Formal analysis, Project administration, Validation, Investigation, Funding acquisition, Resources, Visualization. AS: Writing – original draft, Writing – review & editing, Methodology, Supervision, Conceptualization, Formal analysis, Project administration, Validation, nvestigation, Funding acquisition, Resources, Visualization. CC: Writing – original draft, Writing – review & editing, Data curation, Methodology, Conceptualization, Formal analysis, Validation, Funding acquisition, Resources, Visualization, Software. JH: Writing – original draft, Writing – review & editing, Methodology, Supervision, Conceptualization, Funding acquisition.

## Footnotes

^1^Microsoft Office 365 EXCEL.

^2^IBM SPSS Statistics for Macintosh, Version 28.0.

^3^ATLAS.ti Scientific Software Development GmbH [ATLAS.ti 22 Mac]. (2022). Retrieved from https://atlasti.com

## References

[ref1] Agis K. Castellanos P. Dacunto F. (2023). How to harness the potential of YouTube's musical universe. Think with Google. Available at: https://www.thinkwithgoogle.com/intl/es-419/insights/tendencias-de-consumo/potencial-musica-youtube/

[ref2] Álvarez Álvarez P. (2022). Analysis of the influence of TikTok on the promotion, distribution and consumption of music. [Thesis project]. Comillas Pontifical University. Available at: http://hdl.handle.net/11531/55796

[ref3] Álvarez-Gayou J. L. (2004). How to perform qualitative research fundamentals and methodology. Barcelona: Paidós Educador.

[ref6] Castañeda Mª B Cabrera A. F. Navarro Y. De Vries W. (2010). Data processing and statistical analysis using SPSS. Porto Alegre: EdiPUCRS.

[ref9] De Aguilera-Moyano M. Adell-Pitarch J. Borges-Rey E. (2010). Imaginative appropriations of music in the new communicative scenarios. Communicate 17, 35–44. doi: 10.3916/C34-2010-02-03

[ref10] del De Moya Martínez M. V. Hernández Bravo J. A. Hernández Bravo J. R. Cózar Gutiérrez R. (2014). Musical uses and attitudes of young people through IT. Music and Education Journal 97, 42–52. Available at: https://www.researchgate.net/publication/352947940_Usos_y_actitudes_musicales_de_los_jovenes_a_traves_de_las_TIC

[ref11] del De Moya Martínez M. V. Segura Tornero A. del Robles de Moya M. V. Bravo Marín R. (2023). Music, technology and social media: Habits and benefits. Arts and Health. Quality of life in social and educational environments. (29–38). Dykinson, S.L. Available at: https://www.digitaliapublishing.com/a/127268/artes-y-salud.-calidad-de-vida-en-los-entornos-sociales-y-educativos

[ref12] Fernández A. M. (2018). The emotional self-regulation of youth through music. Scene. J. Arts 79, 25–57. Available at: https://www.redalyc.org/journal/5611/561159309015/

[ref14] Fustinoni O. (2021). Brain and music: Emotion, creation and interpretation. Buenos Aires: El Ateneo.

[ref15] Galeano S. (2023). What are the social media with the most users across the world (2023). Marketing 4 Ecommerce. Available at: https://marketing4ecommerce.net/cuales-redes-sociales-con-mas-usuarios-mundo-ranking/

[ref17] Gozálvez-Pérez V. Cortijo-Ruíz G. (2023). Human development and social media in digital societies. Sophia Philos. Educ. Collect. 34, 41–64. doi: 10.17163/soph.n34.2023.01

[ref18] Hütt Herrera H. (2012). Social media: a new dissemination tool. Reflections 91, 121–128. Available at: https://revistas.ucr.ac.cr/index.php/reflexiones/article/view/1513/1521

[ref20] Llanga Vargas E. F. Insuasti Cárdenas J. P. (2019). The influence of music on learning. Atlante Journal: Handbooks on Education and Development. 1–11. Available at: https://www.eumed.net/rev/atlante/2019/06/musica-aprendizaje.html

[ref21] Magaudda P. (2012). Objects to listen to: Hifi, iPod and consumption of musical technologies: Il Mulino.

[ref23] Marquez I. V. (2011). Music and experience: from primitive societies to social media. Aibr. Ibero-American. Anthropol. J. 6, 193–214. Available at: https://www.redalyc.org/articulo.oa?id=62322211004

[ref26] Moloch H. (2003). Fenomenologia del tostapane. Rafaello Cortina: Come gli oggetti quotidiani diventano ciò che sono.

[ref28] Moscoso Córdova G. V. Espín Pastor V. E. Ortiz Villalba P. G. del Caiza Vega M. R. Zabala Peñaloza E. J. (2021). Efectos de la música en el rendimiento funcional de universitarios. Mediciencias Uta 5, 157–161. doi: 10.31243/mdc.uta.v5i4.1.1161.2021

[ref29] Mosquera Cabrera I. (2013). Influence of music on emotions: a brief review. Realitas J. Soc. Hum. Sci. Arts 1, 34–38. Available at: https://dialnet.unirioja.es/servlet/articulo?codigo=4766791

[ref30] Otzen T. Manterola C. (2017). Sampling techniques for a study population. Int. J. Morphol. 35, 227–232. doi: 10.4067/S0717-95022017000100037

[ref31] Oudshoorn N. Pinch T. (2003). How users matter. The Co-Construction of Users and Technology: The MIT Press Available at: http://hdl.handle.net/2027/spo.3310410.0008.110.

[ref32] Redacción La. (2023). WhatsApp, Facebook, Instagram and YouTube lead the use of social media in Spain. PublicidAD Newspaper. Available at: https://lapublicidad.net/whatsapp-facebook-instagram-y-youtube-lideran-el-uso-de-redes-sociales-en-espana/

[ref33] Sampieri R. Collado C. y Lucio P. (2003). Research methodology. Available at: http://metodos-comunicacion.sociales.uba.ar/wp-content/uploads/sites/219/2014/04/Hernandez-Sampieri-Cap-1.pdf

[ref34] Shuttleworth M. (2019). Descritive research design. Available at: https://explorable.com/es/diseno-de-investigacion-descriptiva

[ref35] Soria Urios G. García Moreno J. M. Duque P. (2011). Music and brain (II): evidence of musical training in the brain. J. Neurol. 53, 739–746. doi: 10.33588/rn.5312.2011475 22127661

[ref36] Tejada Garitano E. Castaño Garrido C. Romero Andonegui A. (2019). Habits of use within the social circles of pre-adolescents. IJDE 22, 119–133. doi: 10.5944/ried.22.2.23245

[ref37] Torres Lazcano L. E. (2016). Music as an alternative Media for Communication Linked to the revolution and social reconfiguration [thesis]. Autonomous University of Mexico State. Available at: http://ri.uaemex.mx/handle/20.500.11799/49199

[ref39] Uribe P. (2013). Music and social media. Arts in social media (pp. 87-98). Facultad de Filosofía Universidad Nacional Autónoma de México. Estudio Paraíso. Available at: http://hdl.handle.net/10391/4940

[ref40] Vázquez Chas L. (2023). Online social media as buffers against loneliness during lockdown. SEECI Commun. J. 56, 249–264. doi: 10.15198/seeci.2023.56.e828

[ref41] Vela A. A. (2020). Changes in consumption and the music corporation until the arrival of Spotify. Amaral case study. [thesis project]. University of Zaragoza. Available at: https://zaguan.unizar.es/record/94693

[ref42] Warner T. (2017). Technology and creativity: Trevor horn and digital revolution. Taylor & Francis Group, London: Routledge.

[ref43] Wortman A. (2019). Between standardization and individualization. Internet, digital platforms and musical preferences of CABA adolescents. Hypertexts 7, 200–224. Available at: https://revistas.unlp.edu.ar/hipertextos/article/view/7955

