# Supplementary material for: The population of Veneto faced with technological consumption of music during the pandemic
Source: Front Psychol. 2024 Jul 8;15:1329531. doi: 10.3389/fpsyg.2024.1329531 (PMC11262052; doi:10.3389/fpsyg.2024.1329531)

- 1. ***Calibration responses***

| **Q1:***Quanti anni hai?* | | | | | |
| --- | --- | --- | --- | --- | --- |
|  | | Frequency | Percentage | Valid percentage | Accumulated percentaje |
|  | 18-24 | 79 | 23,4 | 23,4 | 23,4 |
|  | 25-34 | 34 | 10,1 | 10,1 | 33,4 |
|  | 35-44 | 36 | 10,7 | 10,7 | 44,1 |
|  | 45-54 | 39 | 11,5 | 11,5 | 55,6 |
|  | 55-64 | 89 | 26,3 | 26,3 | 82,0 |
|  | 65 o più | 59 | 17,5 | 17,5 | 99,4 |
|  | NC | 2 | ,6 | ,6 | 100,0 |
|  | Total | 338 | 100,0 | 100,0 |  |

Table 1. Age

Figure 1. Age range


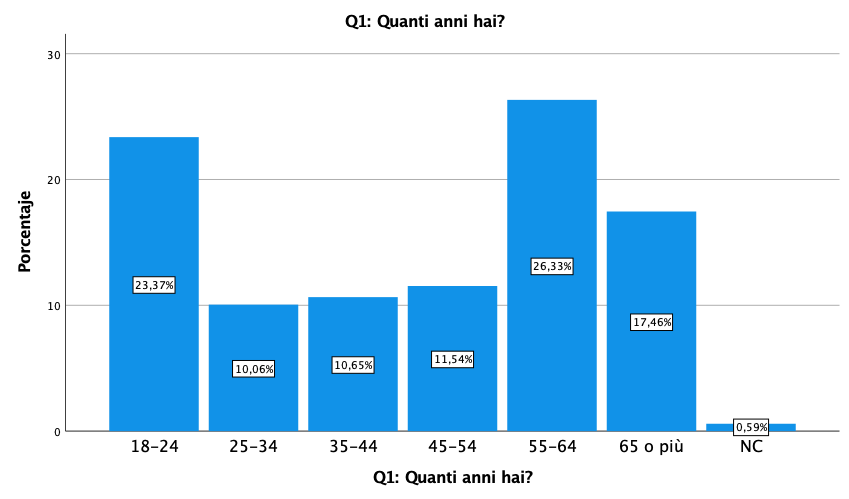


| **Q2:***Qual è il vostro sesso?* | | | | | | |
| --- | --- | --- | --- | --- | --- | --- |
|  | | Frequency | | Percentage | Valid percentage | Accumulated percentaje |
|  | Donna | | 192 | 56,8 | 56,8 | 56,8 |
|  | Uomo | | 144 | 42,6 | 42,6 | 99,4 |
|  | NC | | 2 | ,6 | ,6 | 100,0 |
|  | Total | | 338 | 100,0 | 100,0 |  |

Table 2. Sex

Figure 2. Sex


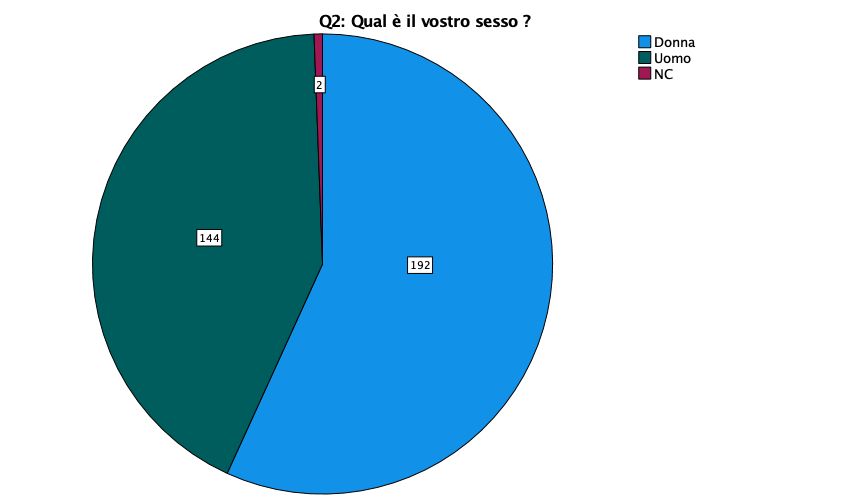


Table 3. Relationships years sex

| *Q1: Quanti anni hai? / Q2: Qual è il vostro sesso?* | | | | | |
| --- | --- | --- | --- | --- | --- |
|  | | *Q2* | | | Total |
|  |  | Donna | Uomo | NC |  |
| *Q1* | 18-24 | 57 | 21 | 1 | 79 |
|  | 25-34 | 18 | 16 | 0 | 34 |
|  | 35-44 | 21 | 15 | 0 | 36 |
|  | 45-54 | 16 | 23 | 0 | 39 |
|  | 55-64 | 52 | 37 | 0 | 89 |
|  | 65 o più | 27 | 32 | 0 | 59 |
|  | NC | 1 | 0 | 1 | 2 |
| Total | | 192 | 144 | 2 | 338 |

Figure 3. Relationships years sex


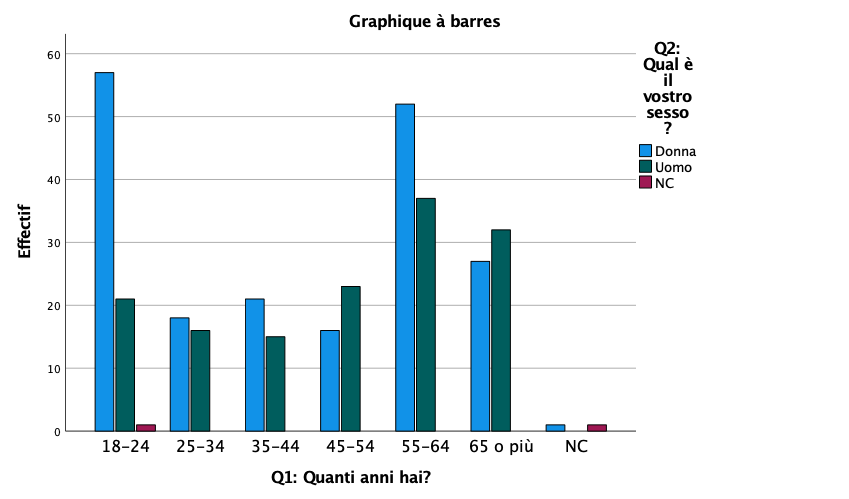


Table 4. Professional partner category

| **Q3:***Qual è la sua occupazione?* | | | | | |
| --- | --- | --- | --- | --- | --- |
|  | | Frequency | Percentage | Valid percentage | Accumulated percentaje |
|  | Studente di laurea triennale | 72 | 21,3 | 21,3 | 21,3 |
|  | Studente di laurea magistrale | 12 | 3,6 | 3,6 | 24,9 |
|  | Studente di dottorato | 5 | 1,5 | 1,5 | 26,3 |
|  | Studente di altri corsi/master | 4 | 1,2 | 1,2 | 27,5 |
|  | Lavoratore dipendente | 173 | 51,2 | 51,2 | 78,7 |
|  | Disoccupato | 11 | 3,3 | 3,3 | 82,0 |
|  | Pensionato | 59 | 17,5 | 17,5 | 99,4 |
|  | NC | 2 | ,6 | ,6 | 100,0 |
|  | Total | 338 | 100,0 | 100,0 |  |

Table 5. Professional partner category

| *Q3: Qual è la sua occupazione? / Q2: Qual è il vostro sesso?* | | | | | |
| --- | --- | --- | --- | --- | --- |
|  | | *Q2* | | | Total |
|  |  | Donna | Uomo | NC |  |
| *Q3* | Studente di laurea triennale | 57 | 15 | 0 | 72 |
|  | Studente di laurea magistrale | 5 | 7 | 0 | 12 |
|  | Studente di dottorato | 2 | 2 | 1 | 5 |
|  | Studente di altri corsi/master | 3 | 1 | 0 | 4 |
|  | Lavoratore dipendente | 87 | 86 | 0 | 173 |
|  | Disoccupato | 7 | 4 | 0 | 11 |
|  | Pensionato | 31 | 27 | 1 | 59 |
|  | NC | 0 | 2 | 0 | 2 |
| Total | | 192 | 144 | 2 | 338 |

Figure 4. Professional partner category based on sex


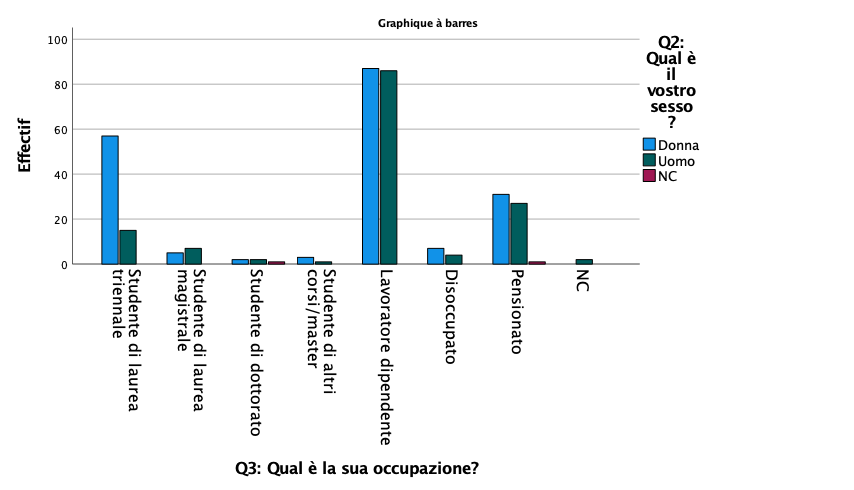


Figure 5. People in each category


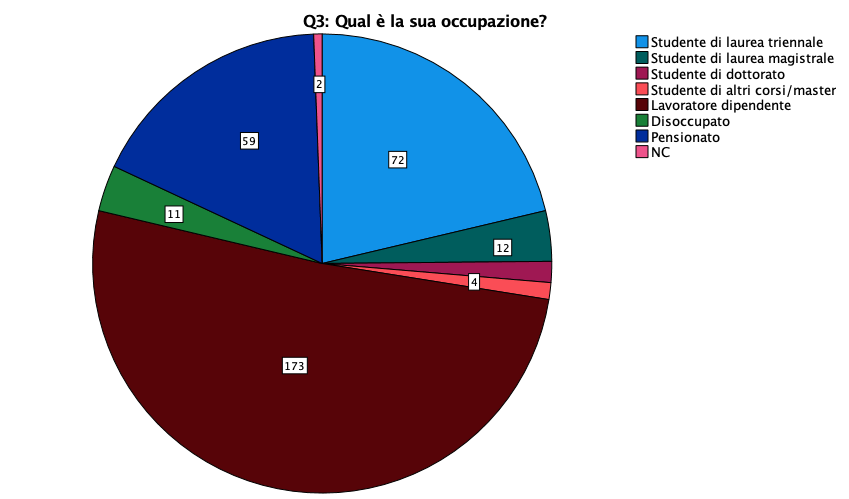


Table 6. Place of residence

| **Q4:***In che provincia o città abiti ?* | | | | | |
| --- | --- | --- | --- | --- | --- |
|  | | Frequency | Percentage | Valid percentage | Accumulated percentaje |
|  | Belluno | 5 | 1,5 | 1,5 | 1,5 |
|  | Padova | 215 | 63,6 | 63,6 | 65,1 |
|  | Rovigo | 2 | ,6 | ,6 | 65,7 |
|  | Treviso | 22 | 6,5 | 6,5 | 72,2 |
|  | Venezia | 31 | 9,2 | 9,2 | 81,4 |
|  | Verona | 9 | 2,7 | 2,7 | 84,0 |
|  | Vicenza | 32 | 9,5 | 9,5 | 93,5 |
|  | NC | 22 | 6,5 | 6,5 | 100,0 |
|  | Total | 338 | 100,0 | 100,0 |  |

Figure 6. Circular representation of the place of residence


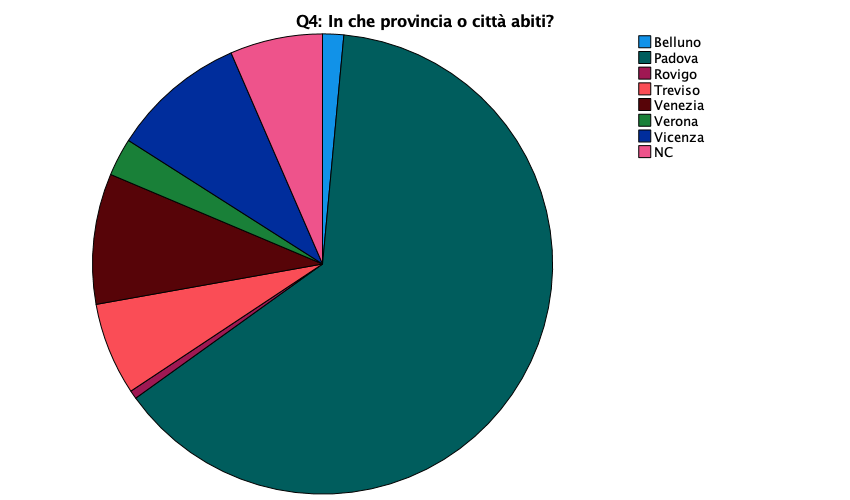


- 1. ***Quantitative answers***

Figure 7. Graphic representation of changes during confinement


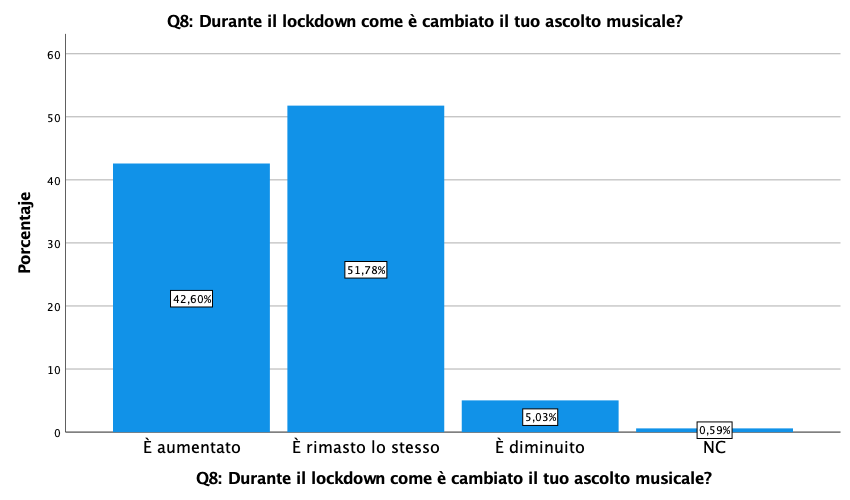


Table 7. Variation of musical listening

| **Q9:** *Quanto è variato l'ascolto musicale?* | | | | | |
| --- | --- | --- | --- | --- | --- |
|  | | Frequency | Percentage | Valid percentage | Cumulative percentage |
|  | L'ascolto è variato poco | 216 | 63,9 | 63,9 | 63,9 |
|  | L'ascolto è variato abbastanza | 83 | 24,6 | 24,6 | 88,5 |
|  | L'ascolto è variato molto | 32 | 9,5 | 9,5 | 97,9 |
|  | NC | 7 | 2,1 | 2,1 | 100,0 |
|  | Total | 338 | 100,0 | 100,0 |  |

Figure 8. Circular representation of these variations


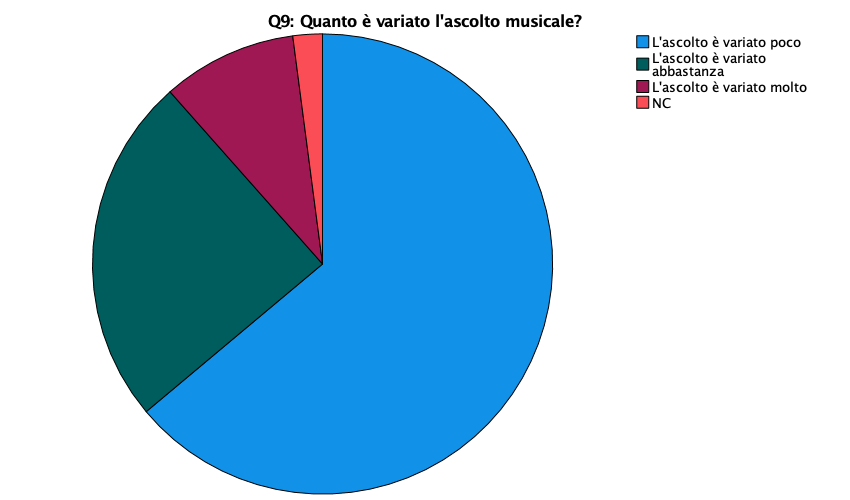


Table 8. Changes in musical habits after the pandemic

| **Q11:** *Ritieni che dopo la pandemia il tuo ascolto musicale sia cambiato?* | | | | | |
| --- | --- | --- | --- | --- | --- |
|  | | Frequency | Percentage | Valid percentage | Cumulative percentage |
|  | Sì, è aumentato | 119 | 35,2 | 35,2 | 35,2 |
|  | No, è lo stesso | 204 | 60,4 | 60,4 | 95,6 |
|  | Sì, è diminuito | 15 | 4,4 | 4,4 | 100,0 |
|  | Total | 338 | 100,0 | 100,0 |  |

Figure 9. Representation of these changes in habits


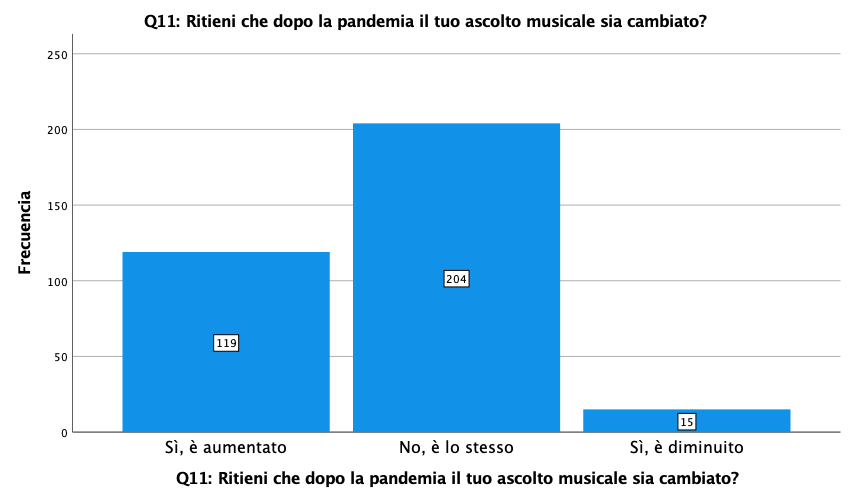


Table 9. Increased musical listening

| ***Estadísticas*** | | | | | | |
| --- | --- | --- | --- | --- | --- | --- |
|  | | **Q11_1:***Ora lo apprezzo di più* | | **Q11_2:***Ho conosciuto nuovi artisti* | **Q11_3:***Ho scoperto nuovi generi musicali* | **Q11_4:***Do più importanza alle piccole cose, compreso l'ascolto della musica* |
| N | Validez | | 119 | 119 | 119 | 119 |
|  | Casos perdidos | | 0 | 0 | 0 | 0 |

Table 10. Change in enjoying music

| **Q11_1:** *Ora lo apprezzo di più* | | | | | |
| --- | --- | --- | --- | --- | --- |
|  | | Frequency | Percentage | Valid percentage | Cumulative percentage |
|  | 1 | 7 | 5,9 | 5,9 | 5,9 |
|  | 2 | 4 | 3,4 | 3,4 | 9,2 |
|  | 3 | 15 | 12,6 | 12,6 | 21,8 |
|  | 4 | 31 | 26,1 | 26,1 | 47,9 |
|  | 5 | 17 | 14,3 | 14,3 | 62,2 |
|  | NC | 45 | 37,8 | 37,8 | 100,0 |
| Total | | 119 | 100,0 | 100,0 |  |

Figure 10. Assessment of enjoying music


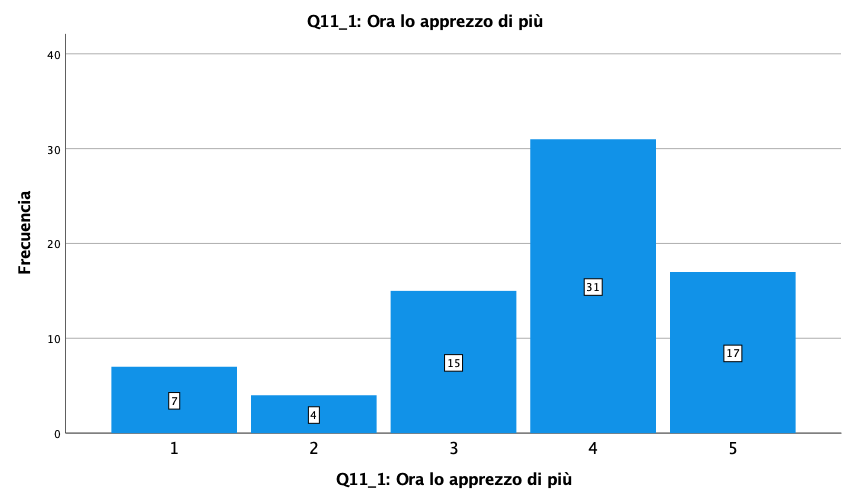


Table 11. Meet new artists

| **Q11_2:** *Ho conosciuto nuovi artista* | | | | | |
| --- | --- | --- | --- | --- | --- |
|  | | Frequency | Percentage | Valid percentage | Cumulative percentage |
|  | 1 | 1 | ,8 | ,8 | 0,8 |
|  | 2 | 10 | 8,4 | 8,4 | 9,2 |
|  | 3 | 23 | 19,3 | 19,3 | 28,5 |
|  | 4 | 23 | 19,3 | 19,3 | 47,9 |
|  | 5 | 22 | 18,5 | 18,5 | 66,4 |
|  | NC | 40 | 33,6 | 33,6 | 100,0 |
| Total | | 119 | 100,0 | 100,0 |  |

Figure 11. Meet new artists


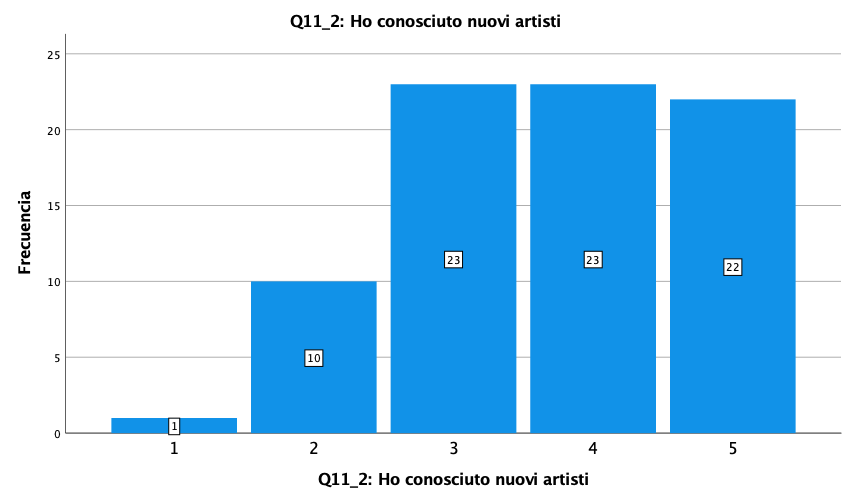


Table 12. Discover a new musical genre

| **Q11_3:** *Ho scoperto nuovi generi musicali* | | | | | |
| --- | --- | --- | --- | --- | --- |
|  | | Frequency | Percentage | Valid percentage | Cumulative percentage |
| Validez | 1 | 10 | 8,4 | 8,4 | 8,4 |
|  | 2 | 12 | 10,1 | 10,1 | 18,5 |
|  | 3 | 23 | 19,3 | 19,3 | 37,8 |
|  | 4 | 17 | 14,3 | 14,3 | 52,1 |
|  | 5 | 14 | 11,8 | 11,8 | 63,9 |
|  | Total | 43 | 36,1 | 36,1 | 100,0 |
| Total | | 119 | 100,0 | 100,0 |  |

Figure 12. Assessment a new musical genre


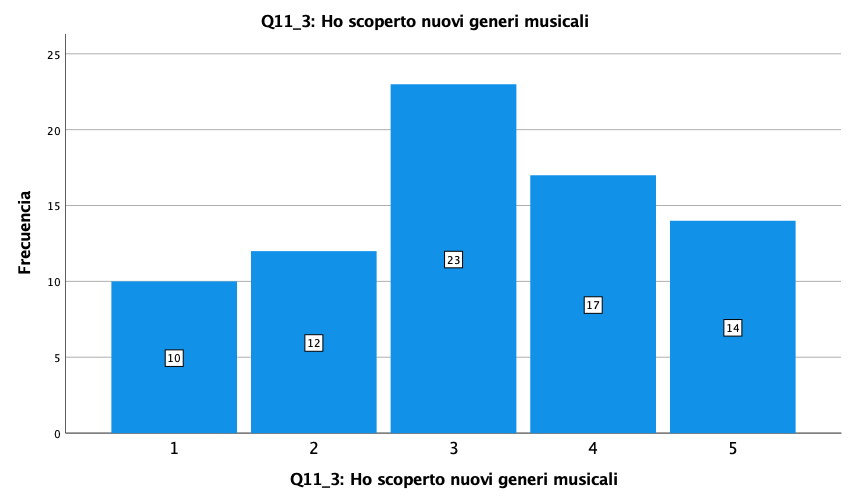


Table 13. More important listen to music

| **Q11_4:** *Do più importanza alle piccole cose, compreso l'ascolto della música* | | | | | |
| --- | --- | --- | --- | --- | --- |
|  | | Frequency | Percentage | Valid percentage | Cumulative percentage |
| Validez | 1 | 6 | 5,0 | 5,0 | 5,0 |
|  | 2 | 11 | 9,2 | 9,2 | 14,2 |
|  | 3 | 18 | 15,1 | 15,1 | 29,3 |
|  | 4 | 24 | 20,2 | 20,2 | 49,5 |
|  | 5 | 23 | 19,3 | 19,3 | 68,9 |
|  | NC | 37 | 31,1 | 31,1 | 100,0 |
| Total | | 119 | 100,0 | 100,0 |  |

Figure 13. Musical listening assessment


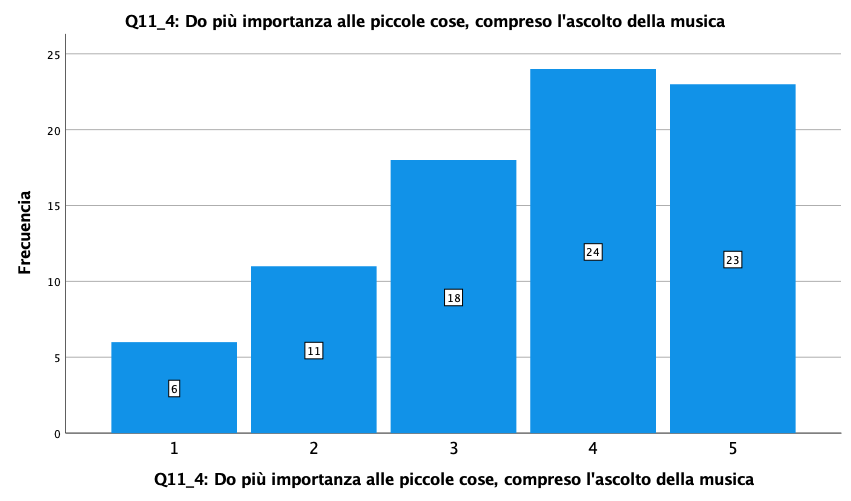


Table 14. Reasons for listening to music

| **Q12:** *Perché ascolti musica?* | | | | | |
| --- | --- | --- | --- | --- | --- |
|  | | Frequency | Percentage | Valid percentage | Cumulative percentage |
| **Perché mi piace** | Sì | 268 | 79,3 | 79,3 | 79,3 |
|  | No | 70 | 20,7 | 20,7 | 100,0 |
|  | Total | 338 | 100,0 | 100,0 |  |
| **Perché mi tira su di morale** | Sì | 128 | 37,9 | 37,9 | 37,9 |
|  | No | 210 | 62,1 | 62,1 | 100,0 |
|  | Total | 338 | 100,0 | 100,0 |  |
| **Perché mi intrattiene** | Sì | 136 | 40,2 | 40,2 | 40,2 |
|  | No | 202 | 59,8 | 59,8 | 100,0 |
|  | Total | 338 | 100,0 | 100,0 |  |
| **Perché mi fa compagnia e mi aiuta a non sentirmi solo** | Sì | 116 | 34,3 | 34,3 | 34,3 |
|  | No | 222 | 65,7 | 65,7 | 100,0 |
|  | Total | 338 | 100,0 | 100,0 |  |
| **Perché ci sono abituato** | Sì | 60 | 17,8 | 17,8 | 17,8 |
|  | No | 278 | 82,2 | 82,2 | 100,0 |
|  | Total | 338 | 100,0 | 100,0 |  |
| **Perché è qualcosa di importante nella mia vita quotidiana** | Sì | 173 | 51,2 | 51,2 | 51,2 |
|  | No | 165 | 48,8 | 48,8 | 100,0 |
|  | Total | 338 | 100,0 | 100,0 |  |
| **Perché va di moda** | Sì | 1 | ,3 | ,3 | ,3 |
|  | No | 337 | 99,7 | 99,7 | 100,0 |
|  | Total | 338 | 100,0 | 100,0 |  |
| **Non lo so** | Sì | 8 | 2,4 | 2,4 | 2,4 |
|  | No | 330 | 97,6 | 97,6 | 100,0 |
|  | Total | 338 | 100,0 | 100,0 |  |
| **NC** | NC | 6 | 1,8 | 1,8 | 1,8 |
|  | No | 332 | 98,2 | 98,2 | 100,0 |
|  | Total | 338 | 100,0 | 100,0 |  |

Table 15. Content shared on social networks

| **Q13:** *Hai condiviso le tue registrazioni musicali sui social network durante la quarantena?* | | | | | |
| --- | --- | --- | --- | --- | --- |
|  | | Frequency | Percentage | Valid percentage | Cumulative percentage |
|  | Sì | 72 | 21,3 | 21,3 | 21,3 |
|  | No | 253 | 74,9 | 74,9 | 96,2 |
|  | NC | 13 | 3,8 | 3,8 | 100,0 |
|  | Total | 338 | 100,0 | 100,0 |  |

Figure 14. Circular representation of shared content


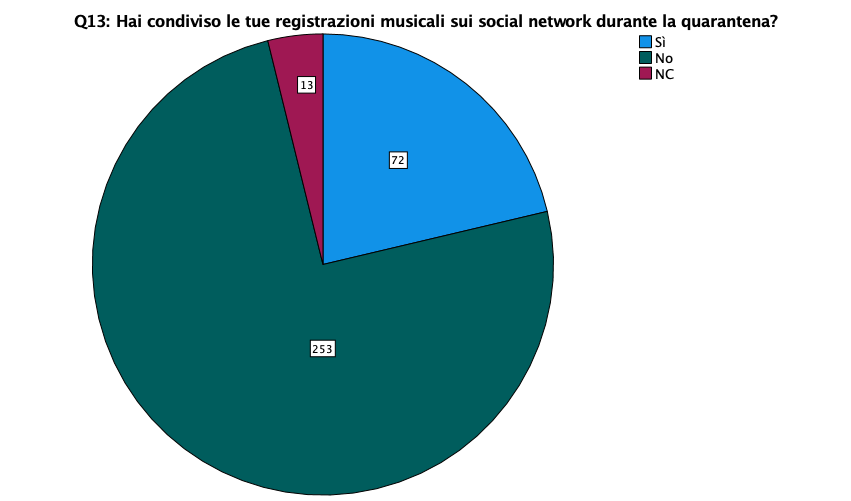


Table 16. Difficulties during confinement

| **Q14:** *Qual è stata durante l'ultima quarantena la cosa più difficile per te? Scegli una risposta o indica "altro":* | | | | | | | | | |
| --- | --- | --- | --- | --- | --- | --- | --- | --- | --- |
|  | | Frequency | | Percentage | | Valid percentage | | Cumulative percentage | |
|  | Isolamento dalla famiglia e dagli amici | | 121 | | 35,8 | | 35,8 | | 35,8 |
|  | paura/insicurezza | | 73 | | 21,6 | | 21,6 | | 57,4 |
|  | problemi economici | | 18 | | 5,3 | | 5,3 | | 62,7 |
|  | noia | | 55 | | 16,3 | | 16,3 | | 79,0 |
|  | altro | | 61 | | 18,0 | | 18,0 | | 97,0 |
|  | NC | | 10 | | 3,0 | | 3,0 | | 100,0 |
|  | Total | | 338 | | 100,0 | | 100,0 | |  |

Figure 15. Representation of difficulties during lockdown


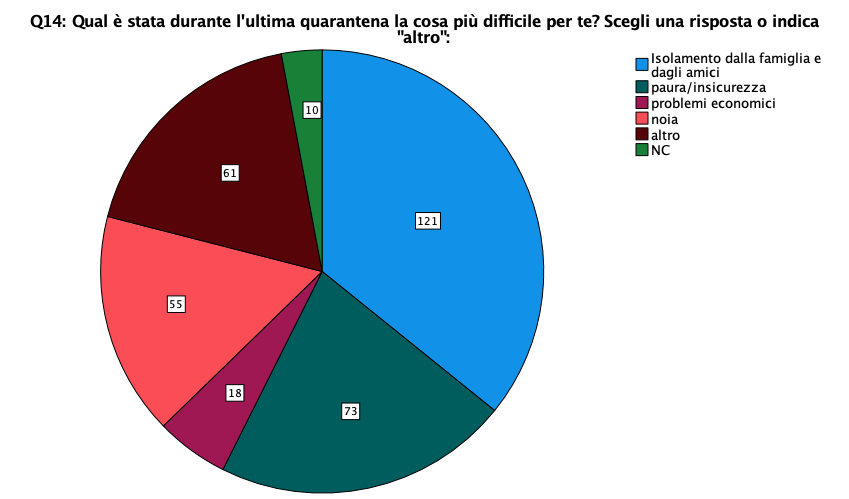


Table 17. Music help

| **Q15:** *In che modo ti ha aiutato la musica durante la quarantena?* | | | | | |
| --- | --- | --- | --- | --- | --- |
|  | | Frequency | Percentage | Valid percentage | Cumulative percentage |
| **Per riposare** | Sì | 71 | 21,0 | 21,0 | 21,0 |
|  | No | 267 | 79,0 | 79,0 | 100,0 |
|  | Total | 338 | 100,0 | 100,0 |  |
| **Mi ha migliorato l'umore** | Sì | 213 | 63,0 | 63,0 | 63,0 |
|  | No | 125 | 37,0 | 37,0 | 100,0 |
|  | Total | 338 | 100,0 | 100,0 |  |
| **Per lavorare** | Sì | 47 | 13,9 | 13,9 | 13,9 |
|  | No | 291 | 86,1 | 86,1 | 100,0 |
|  | Total | 338 | 100,0 | 100,0 |  |
| **Mi ha donato soddisfazione** | Sì | 78 | 23,1 | 23,1 | 23,1 |
|  | No | 260 | 76,9 | 76,9 | 100,0 |
|  | Total | 338 | 100,0 | 100,0 |  |
| **La musica è stata un mezzo per relazionarmi con altre persone** | Sì | 37 | 10,9 | 10,9 | 10,9 |
|  | No | 301 | 89,1 | 89,1 | 100,0 |
|  | Total | 338 | 100,0 | 100,0 |  |
| **NC** | NC | 21 | 6,2 | 6,2 | 6,2 |
|  | No | 317 | 93,8 | 93,8 | 100,0 |
|  | Total | 338 | 100,0 | 100,0 |  |

Table 18. Messages on social networks

| **Q16:** *Indica le tue preferenze sui contenuti dei social media durante il lockdown?* | | | | | | |
| --- | --- | --- | --- | --- | --- | --- |
|  | | Frequency | | Percentage | Valid percentage | Cumulative percentage |
| **Messaggi di speranza** | Sì | | 74 | 21,9 | 21,9 | 21,9 |
|  | No | | 264 | 78,1 | 78,1 | 100,0 |
|  | Total | | 338 | 100,0 | 100,0 |  |
| **Anteprime musicali** | Sì | | 44 | 13,0 | 13,0 | 13,0 |
|  | No | | 294 | 87,0 | 87,0 | 100,0 |
|  | Total | | 338 | 100,0 | 100,0 |  |
| **Temi preferiti** | Sì | | 102 | 30,2 | 30,2 | 30,2 |
|  | No | | 236 | 69,8 | 69,8 | 100,0 |
|  | Total | | 338 | 100,0 | 100,0 |  |
| **Classici o temi di sempre** | Sì | | 157 | 46,4 | 46,4 | 46,4 |
|  | No | | 181 | 53,6 | 53,6 | 100,0 |
|  | Total | | 338 | 100,0 | 100,0 |  |
| **NC** | NC | | 32 | 9,5 | 9,5 | 9,5 |
|  | No | | 306 | 90,5 | 90,5 | 100,0 |
|  | Total | | 338 | 100,0 | 100,0 |  |

Table 19. Importance of music in lockdown

| **Q17:** *Quanto è stata importante per te la musica durante il lockdown? (da 1, che equivale a nulla, a 5)* | | | | | |
| --- | --- | --- | --- | --- | --- |
|  | | Frequency | Percentage | Valid percentage | Cumulative percentage |
|  | 1 | 17 | 5,0 | 5,0 | 5,0 |
|  | 2 | 34 | 10,1 | 10,1 | 15,1 |
|  | 3 | 93 | 27,5 | 27,5 | 42,6 |
|  | 4 | 92 | 27,2 | 27,2 | 69,8 |
|  | 5 | 97 | 28,7 | 28,7 | 98,5 |
|  | NC | 5 | 1,5 | 1,5 | 100,0 |
| Total | | 338 | 100,0 | 100,0 |  |

Figure 16. Representation of the importance of music


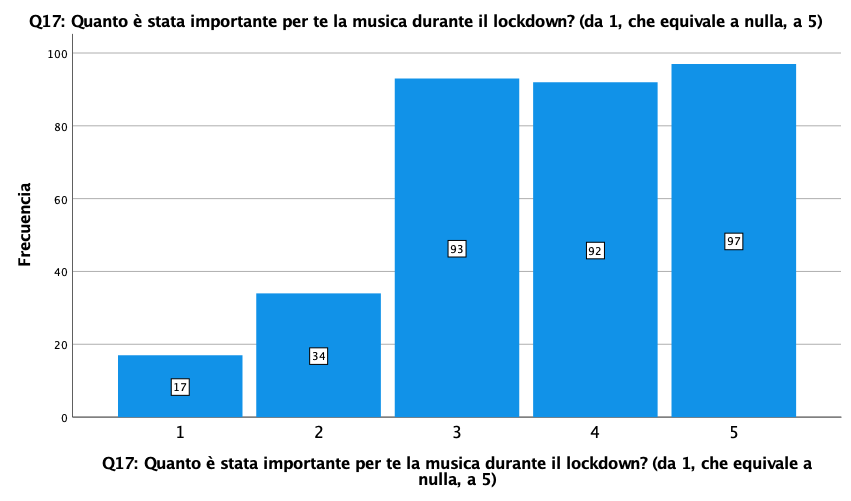


Table 20. Musical works

| **Q18:** *Hai ascoltato o visto opere musicali durante la quarantena?* | | | | | |
| --- | --- | --- | --- | --- | --- |
|  | | Frequency | Percentage | Valid percentage | Cumulative percentage |
|  | Sì | 139 | 41,1 | 41,1 | 41,1 |
|  | No | 192 | 56,8 | 56,8 | 97,9 |
|  | NC | 7 | 2,1 | 2,1 | 100,0 |
|  | Total | 338 | 100,0 | 100,0 |  |

Figure 17. Circular representation of the view of musical works


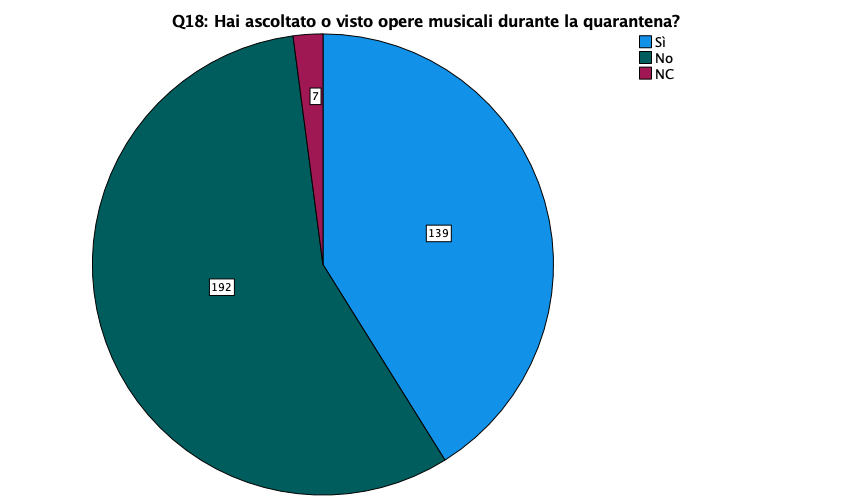


1. **Music and physical activity**

Table 21. Physical activity

| **Q19:** *Fai esercizio fisico?* | | | | | |
| --- | --- | --- | --- | --- | --- |
|  | | Frequency | Percentage | Valid percentage | Cumulative percentage |
| Validez | Sì | 221 | 65,4 | 65,4 | 65,4 |
|  | No | 111 | 32,8 | 32,8 | 98,2 |
|  | NC | 6 | 1,8 | 1,8 | 100,0 |
|  | Total | 338 | 100,0 | 100,0 |  |

Figure 18. Circular representation of physical activity


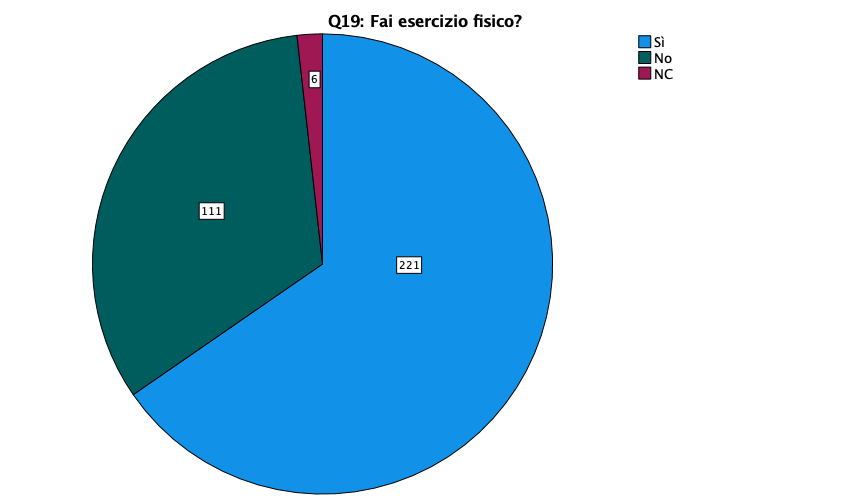


Table 22. Weekly frequency

| **Q19_1:** *Se sì, quanti giorni alla settimana?* | | | | | |
| --- | --- | --- | --- | --- | --- |
|  | | Frequency | Percentage | Valid percentage | Cumulative percentage |
| Validez | Un giorno | 19 | 5,6 | 5,6 | 5,6 |
|  | due giorni | 63 | 18,6 | 18,6 | 24,2 |
|  | tre giorni | 74 | 21,9 | 21,9 | 46,1 |
|  | quattro giorni | 25 | 7,4 | 7,4 | 53,5 |
|  | cinque giorni | 22 | 6,5 | 6,5 | 60 |
|  | sei giorni | 6 | 1,8 | 1,8 | 61,8 |
|  | ogni giorno | 36 | 10,7 | 10,7 | 72,5 |
|  | altra risposta | 10 | 3,0 | 3,0 | 75,4 |
|  | NC | 83 | 24,6 | 24,6 | 100,0 |
| Total | | 338 | 100,0 | 100,0 |  |

Figure 19. Circular representation of the frequency per week


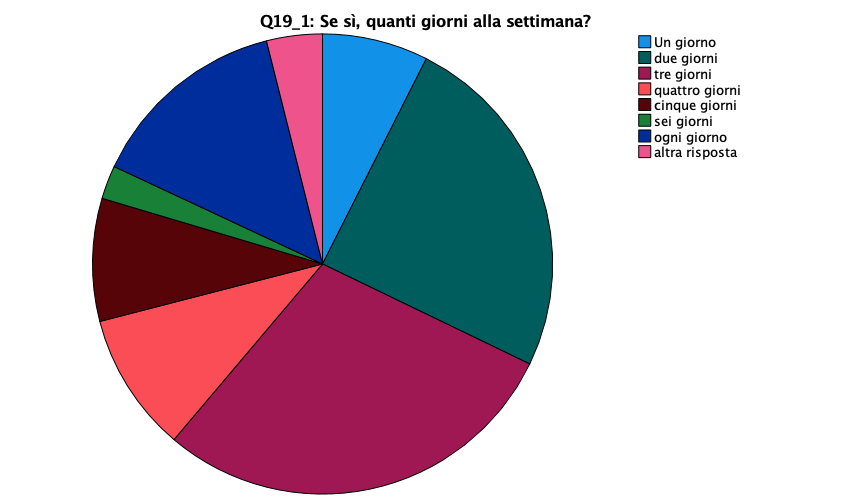


Table 23. Daily frequency

| **Q19_2:** *Quante ore al giorno?* | | | | | |
| --- | --- | --- | --- | --- | --- |
|  | | Frequency | Percentage | Valid percentage | Cumulative percentage |
|  | meno di un'ora | 53 | 15,7 | 15,7 | 15,7 |
|  | 1 ora | 121 | 35,8 | 35,8 | 51,5 |
|  | un'ora e mezza | 13 | 3,8 | 3,8 | 55,3 |
|  | 2 ore | 42 | 12,4 | 12,4 | 67,8 |
|  | due ore e mezza | 2 | ,6 | ,6 | 68,3 |
|  | 3 ore | 4 | 1,2 | 1,2 | 69,5 |
|  | 4 ore | 3 | ,9 | ,9 | 70,2 |
|  | più di quattro ore | 6 | 1,8 | 1,8 | 72,1 |
|  | altre risposte | 4 | 1,2 | 1,2 | 73,4 |
|  | Total | 90 | 26,6 | 26,6 | 100,0 |
| Total | | 338 | 100,0 | 100,0 |  |

Figure 20. Circular representation of the daily frequency


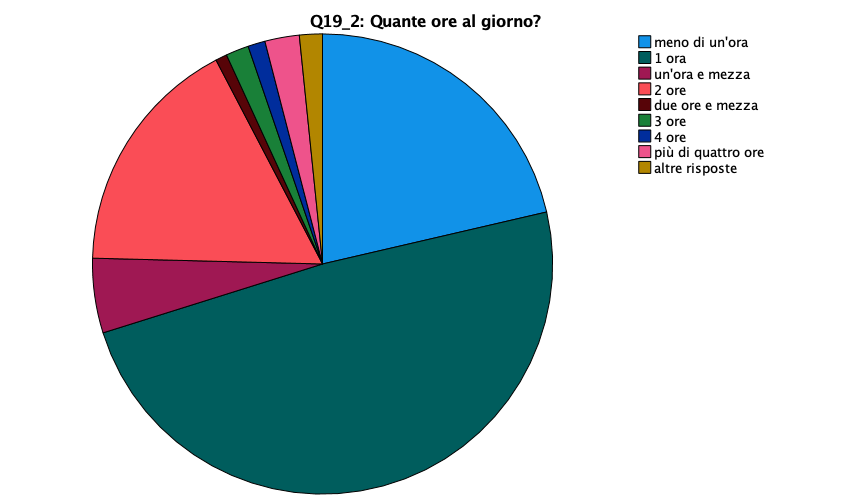


Table 24. Music and physical exercise

| **Q20:** *L’esercizio fisico è accompagnato dalla musica?* | | | | | |
| --- | --- | --- | --- | --- | --- |
|  | | Frequency | Percentage | Valid percentage | Cumulative percentage |
| Validity | Sì | 146 | 43,2 | 43,2 | 43,2 |
|  | No | 131 | 38,8 | 38,8 | 82,0 |
|  | NC | 61 | 18,0 | 18,0 | 100,0 |
|  | Total | 338 | 100,0 | 100,0 |  |

Figure 21. Circular representation of music and physical exercise


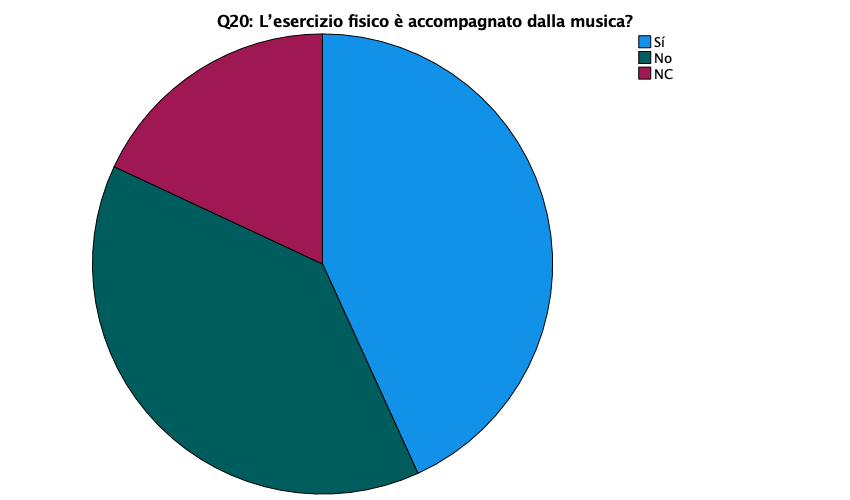


- - 1. **Musical listening**

Table 25. Frequency of weekly music listening

| 1. **Q21:** *Con quale frequenza ascolti musica?* | | | | | |
| --- | --- | --- | --- | --- | --- |
|  | | Frequency | Percentage | Valid percentage | Cumulative percentage |
| Validez | Ogni giorno | 229 | 67,8 | 67,8 | 67,8 |
|  | Cinque giorni a settimana | 37 | 10,9 | 10,9 | 78,7 |
|  | Tre giorni a settimana | 48 | 14,2 | 14,2 | 92,9 |
|  | Meno di una volta alla settimana | 18 | 5,3 | 5,3 | 98,2 |
|  | NC | 6 | 1,8 | 1,8 | 100,0 |
|  | Total | 338 | 100,0 | 100,0 |  |

Figure 22. Representation of weekly listening


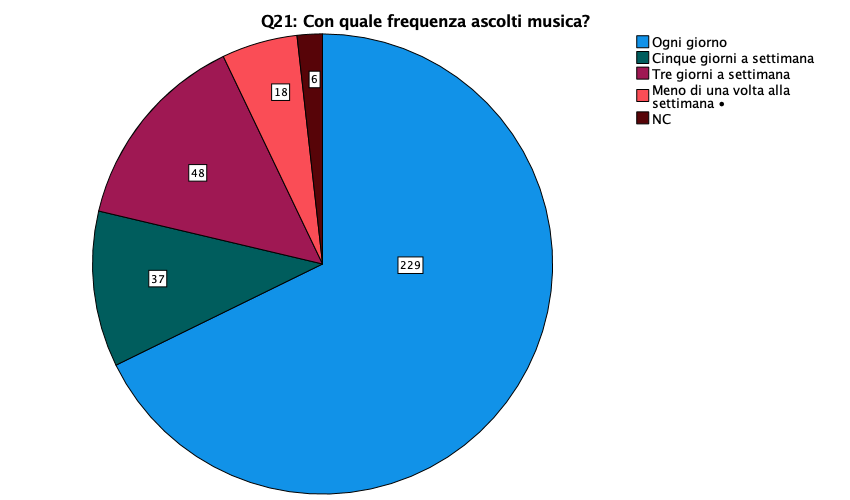


Table 26. Music-related expenses during the pandemic

| **Q22:** *Hai speso soldi per il consumo di musica subito dopo la pandemia? (brani, dischi, album, concerti, abbonamenti a piattaforme...)* | | | | | |
| --- | --- | --- | --- | --- | --- |
|  | | Frequency | Percentage | Valid percentage | Cumulative percentage |
|  | Non ho speso soldi, solo consumo gratuito di musica. | 229 | 67,8 | 67,8 | 67,8 |
|  | 10 euro al mese circa | 66 | 19,5 | 19,5 | 87,3 |
|  | 20 euro al mese circa | 21 | 6,2 | 6,2 | 93,5 |
|  | Più di 20 euro al mese | 17 | 5,0 | 5,0 | 98,5 |
|  | NC | 5 | 1,5 | 1,5 | 100,0 |
|  | Total | 338 | 100,0 | 100,0 |  |

Figure 23. Circular representation of expenses


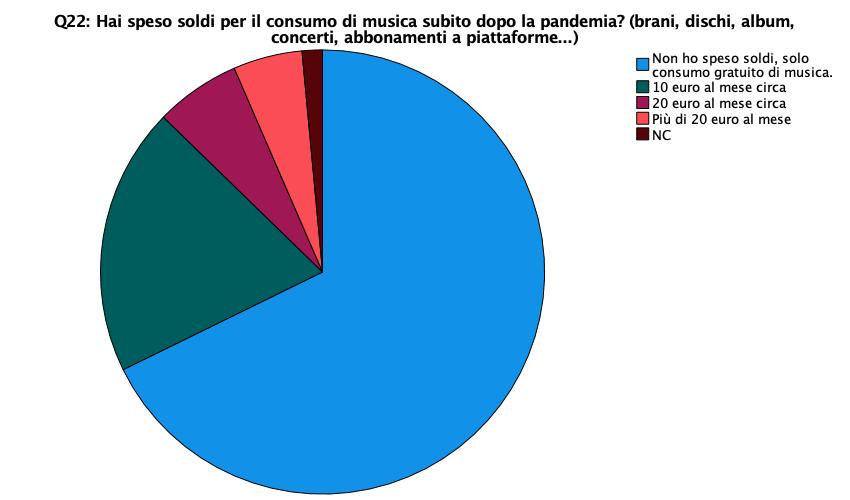


Table 27. Musical genre heard

| **Q23:** *Qual è il genere musicale principale che ascolti?* | | | | | |
| --- | --- | --- | --- | --- | --- |
|  | | Frequency | Percentage | Valid percentage | Cumulative percentage |
| **Musica pop** | Sì | 205 | 60,7 | 60,7 | 60,7 |
|  | No | 133 | 39,3 | 39,3 | 100,0 |
|  | Total | 338 | 100,0 | 100,0 |  |
| **Musica rock and roll** | Sì | 122 | 36,1 | 36,1 | 36,1 |
|  | No | 216 | 63,9 | 63,9 | 100,0 |
|  | Total | 338 | 100,0 | 100,0 |  |
| **Musica classica** | Sì | 117 | 34,6 | 34,6 | 34,6 |
|  | No | 221 | 65,4 | 65,4 | 100,0 |
|  | Total | 338 | 100,0 | 100,0 |  |
| **Musica per bambini** | Sì | 8 | 2,4 | 2,4 | 2,4 |
|  | No | 330 | 97,6 | 97,6 | 100,0 |
|  | Total | 338 | 100,0 | 100,0 |  |
| **Musica jazz** | Sì | 63 | 18,6 | 18,6 | 18,6 |
|  | No | 275 | 81,4 | 81,4 | 100,0 |
|  | Total | 338 | 100,0 | 100,0 |  |
| **Musica dance** | No | 338 | 100,0 | 100,0 | 100,0 |
| **Musica elettronica** | No | 338 | 100,0 | 100,0 | 100,0 |
| **Musica popolare** | No | 338 | 100,0 | 100,0 | 100,0 |
| **Musica rap** | No | 338 | 100,0 | 100,0 | 100,0 |
| **Musica hip-hop** | No | 338 | 100,0 | 100,0 | 100,0 |
| **Altri** | Sì | 124 | 36,7 | 36,7 | 36,7 |
|  | No | 214 | 63,3 | 63,3 | 100,0 |
|  | Total | 338 | 100,0 | 100,0 |  |
| **NC** | NC | 4 | 1,2 | 1,2 | 1,2 |
|  | No | 334 | 98,8 | 98,8 | 100,0 |
|  | Total | 338 | 100,0 | 100,0 |  |

Table 28. Musical formation

| **Q25:** *Che formazione musicale hai?* | | | | | |
| --- | --- | --- | --- | --- | --- |
|  | | Frequency | Percentage | Valid percentage | Cumulative percentage |
|  | Nessuna | 134 | 39,6 | 39,6 | 39,6 |
|  | Livello base | 105 | 31,1 | 31,1 | 70,7 |
|  | Livello medio | 62 | 18,3 | 18,3 | 89,1 |
|  | Livello avanzato | 32 | 9,5 | 9,5 | 98,5 |
|  | NC | 5 | 1,5 | 1,5 | 100,0 |
|  | Total | 338 | 100,0 | 100,0 |  |

Figure 24. Circular representation musical training


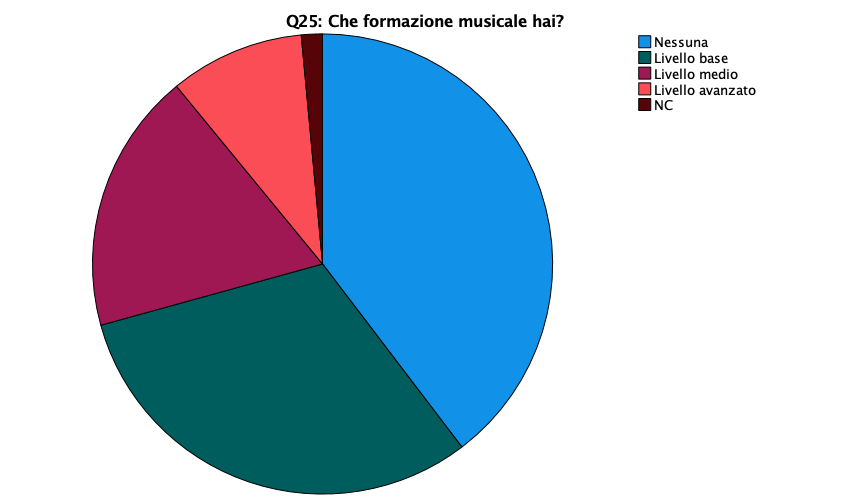

Supplement: Supplementary file 1 [file Data_Sheet_1.docx]
